# Underground trees inhabit varied environmental extremes across the Afrotropics

**DOI:** 10.1093/aob/mcad124

**Published:** 2023-08-29

**Authors:** Anya P Courtenay, Peter W Moonlight, R Toby Pennington, Caroline E R Lehmann

**Affiliations:** GeoSciences, Crew Building, The King’s Buildings, Edinburgh EH9 3FF, UK; Royal Botanic Garden Edinburgh, Edinburgh EH3 5LR, UK; Royal Botanic Garden Edinburgh, Edinburgh EH3 5LR, UK; Botany Department, School of Natural Sciences, Trinity College Dublin, Dublin 2, Ireland; Royal Botanic Garden Edinburgh, Edinburgh EH3 5LR, UK; Geography, University of Exeter, Exeter EX4 4RJ, UK; GeoSciences, Crew Building, The King’s Buildings, Edinburgh EH9 3FF, UK; Royal Botanic Garden Edinburgh, Edinburgh EH3 5LR, UK

**Keywords:** Geoxyle, biogeography, disturbance, frost, fire, herbivory, waterlogging, *Parinari*, *Ozoroa*, *Syzygium*, *Lannea*

## Abstract

**Background and Aims:**

Geoxyles, a distinctive feature of Afrotropical savannas and grasslands, survive recurrent disturbances by resprouting subshrub branches from large below-ground woody structures. Underground trees are a type of geoxyle that independently evolved within woody genera of at least 40 plant families in Africa. The environmental limits and determinants of underground tree biogeography are poorly understood, with the relative influence of frost and fire debated in particular. We aim to quantify variability in the niche of underground tree species relative to their taller, woody tree/shrub congeners.

**Methods:**

Using occurrence records of four Afrotropical genera, *Parinari* (Chrysobalanaceae), *Ozoroa* (Anacardiaceae), *Syzygium* (Myrtaceae) and *Lannea* (Anacardiaceae), and environmental data of nine climate and disturbance variables, the biogeography and niche of underground trees are compared with their open and closed ecosystem congeners.

**Key Results:**

Along multiple environmental gradients and in a multidimensional environmental space, underground trees inhabit significantly distinct and extreme environments relative to open and closed ecosystem congeners. Niche overlap is low among underground trees and their congeners, and also among underground trees of the four genera. Of the study taxa, *Parinari* underground trees inhabit hotter, drier and more seasonal environments where herbivory pressure is greatest. *Ozoroa* underground trees occupy relatively more fire-prone environments, while *Syzygium* underground trees sustain the highest frost frequency and occur in relatively wetter conditions with seasonal waterlogging. *Lannea* underground trees are associated with the lowest temperatures, highest precipitation, and varying exposure to disturbance.

**Conclusions:**

While underground trees exhibit repeated convergent evolution, varied environments shape the ecology and biogeography of this iconic plant functional group. The multiplicity of extreme environments related to fire, frost, herbivory and waterlogging that different underground tree taxa occupy, and the distinctiveness of these environments, should be recognized in the management of African grassy ecosystems.

## INTRODUCTION

Tropical savannas and grasslands support a diversity of specialized growth forms with underground storage organs and below-ground bud banks that facilitate persistence through recurrent and chronic disturbances (Pausas *et al.*, 2018). Among these, plants with the geoxyle growth form conceal long-lived subterranean woody rhizomes, xylopodia or lignotubers from which mostly short-lived and short-stature aerial shoots resprout leaves, inflorescences and fruits between disturbances (White, 1976; Pausas *et al.*, 2018). A substantial number of geoxyles have woody relatives (e.g. Simon *et al.*, 2009) and extensive below-ground woody rhizomatous growth, and have therefore been termed ‘underground trees’ (e.g. White, 1976). Underground trees radiated repeatedly and independently during the late Miocene–Pliocene (5–2.5 Ma) in diverse woody lineages within tropical grassy ecosystems, in both South America (Simon *et al.*, 2009) and Africa (Maurin *et al.*, 2014). Across the Afrotropics, at least 266 underground tree taxa have been recorded within 90 genera across 40 plant families (Maurin *et al.*, 2014). Underground trees, and geoxyles more broadly, have been considered indicators of old-growth savannas and grasslands due to their resprouting capacity (Zaloumis and Bond, 2016; Gomes *et al.*, 2021*a*), contributing to grassy ecosystem biodiversity, functioning, resilience and longevity (Veldman *et al.*, 2015). Understanding of underground trees, a charismatic and ecologically prevalent growth form, remains limited but is important for context-specific management of African grassy ecosystems threatened by anthropogenic pressures such as land conversion and fire suppression (Buisson *et al.*, 2019; Meller *et al.*, 2022; Stevens *et al*., 2022).

**Fig. 1.**
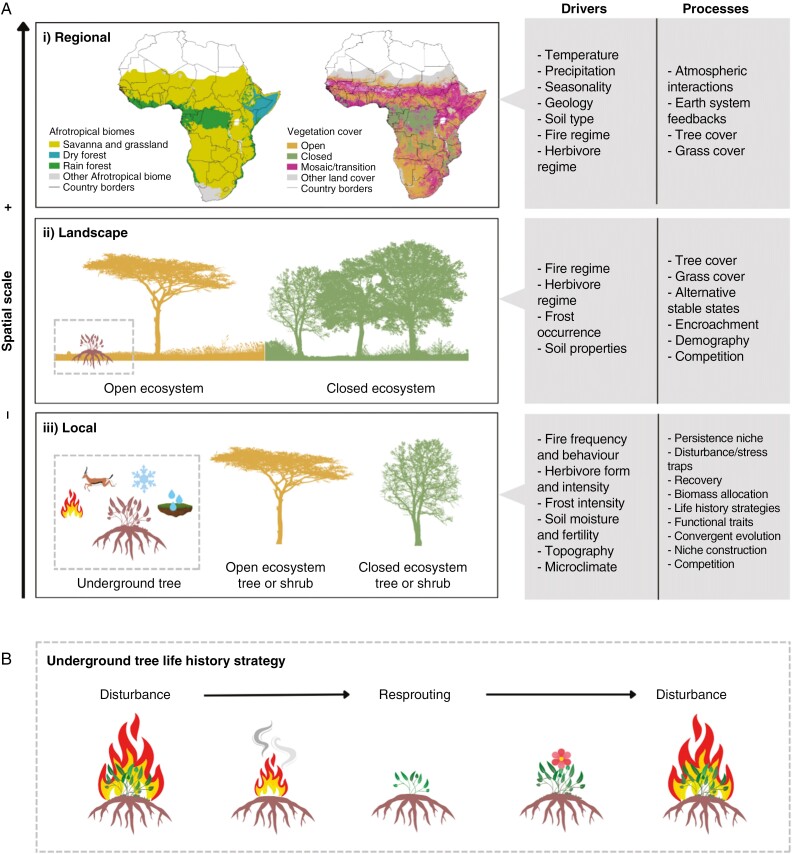
(A) Environmental drivers and processes interact at varying spatial scales, influencing and feeding back over ecological and evolutionary time: (i) across a region (10–10 000 km^2^), climatic, edaphic and some broad-scale disturbances control vegetation distributions [map data for biomes from Pennington *et al*. (2018) and vegetation cover from GlobCover 2009: http://due.esrin.esa.int/page_globcover.php]; (ii) within a landscape (1–10 km^2^), plant communities are primarily determined by environmental stresses that govern vegetation structures, compositions and functions; (iii) locally (<1 km^2^), the specific environmental context, topographic positioning and habitat ecology will influence plant habits, strategies and traits for survival in a particular niche space. This conceptual diagram provides context for how underground trees occupy a more environmentally stressed locale than tree or shrub congeners in open and closed ecosystems. Adapted from Oliveras and Malhi (2016) and based on eco-evolutionary feedbacks outlined by Pausas and Bond (2022). (B) Underground trees persist through disturbance regimes in grassy ecosystems such as fire by resprouting from their below-ground structures and flowering rapidly after. Diagram based on Bond (2016).

Rooted in eco-evolutionary feedbacks (Pausas and Bond, 2022), distributions of underground tree taxa are likely filtered by abiotic and biotic interactions (Fig. 1Ai) that shape vegetation assemblages across heterogeneous landscapes (Fig. 1Aii). Grassy ecosystems are characterized by an open canopy with a continuous shade-intolerant but disturbance-tolerant C_4_ grass ground layer (Bond, 2019), ranging from arid grasslands at boundaries with deserts to mesic savannas at boundaries with closed-canopy forests (Scholes and Archer, 1997; Ratnam *et al.*, 2011; Pennington *et al*., 2018). Underground trees have been recorded across vast environmental gradients, inhabiting a range of open ecosystem settings from suffrutex grasslands on high-elevation plateaus (Zigelski *et al*., 2019*a*) to wetland edges in valley depressions (White, 1976; Zigelski *et al*., 2018), and can occur adjacent to congeneric trees/shrubs from both savanna and forest (Gomes *et al.*, 2019). The niche of an organism describes both its biotic interactions in a community and its abiotic position in an environment, a concept best applied when comparing differences in the functional role of organisms and the physical parameters of habitats they inhabit along environmental gradients (Odum and Barrett, 1971). Local climatic, edaphic, topographic and disturbance conditions coalesce into the niches of underground trees and their congeners (Fig. 1Aiii), also influenced by taxonomically pre-inherited adaptations (Simon and Pennington, 2012; Meller *et al.*, 2022). Underground trees likely occupy a ‘persistence niche’ whereby *in situ* resprouting comes at a trade-off with vertical growth among woody plants (Fig. 1B; Bond and Midgley, 2001). The underground tree growth form is considered advantageous to surviving above-ground survival pressures, and is resilient to disturbance ‘traps’ that otherwise limit woody growth (Maurin *et al.*, 2014; Pennington and Hughes, 2014; Davies *et al.*, 2016).

The relative importance of different eco-evolutionary drivers to the biogeography of underground trees is debated. Disturbance by fire, frost, herbivory and waterlogging have all been mooted as central to the evolution of the growth form in the Afrotropics (White, 1976; Maurin *et al.*, 2014; Davies *et al.*, 2016; Finckh *et al*., 2016; Lamont *et al*., 2017; Meller *et al.*, 2022). Fire is often implicated as the primary driver given the synchronous Miocene evolution of underground trees and expansion of tropical savannas (Simon and Pennington, 2012; Maurin *et al.*, 2014; Pennington and Hughes, 2014). Meanwhile, frost is increasingly recognized as a climatic control on underground trees and wider geoxyles (Finckh *et al*., 2016; Meller *et al.*, 2022), resulting from increased Miocene seasonality (Keeley and Rundel, 2005) and accentuated by topography (Revermann and Finckh, 2013). Debate between fire and frost prevails, but it is worth recognizing that drivers do not necessarily work in isolation (Davies *et al.*, 2016). For example, adaptations initially selected for fire tolerance may prove advantageous in avoiding frost (Lamont *et al*., 2017) or vice versa. Further, herbivory has spatially disparate but comparable impacts to fire as a bottom-up consumer of vegetation (Bond, 2005; Archibald and Hempson, 2016) and has also been suggested as a correlate of geoxyle occurrence, although its influence remains untested (Maurin *et al.*, 2014; Meller *et al.*, 2022). Waterlogged and low-nutrient soils have long been noted to confine some underground trees to the periphery of seasonally inundated wetlands (dambos) (White, 1976), although waterlogging has also not been accounted for in analyses. These environmental drivers have generally been considered as mutually exclusive (Chidumayo, 2019) and the growth form as a functionally homogeneous group, potentially resulting in an oversimplified understanding of underground tree biogeography.

To determine the distinctiveness of underground tree niches, we quantify the environmental distribution of underground trees and their congeneric taxa in both open savanna/grassland ecosystems and closed forest ecosystems of the Afrotropics. Our study takes a genus-level approach to compare environmental occupancy among four well-understood woody genera with underground tree taxa that span a range of environments: *Parinari* (Chrysobalanaceae), *Ozoroa* (Anacardiaceae), *Syzygium* (Myrtaceae) and *Lannea* (Anacardiaceae). Geospatial and statistical analyses of climate, stress and disturbance are used to test whether underground trees have similar or different environmental preferences to their congeners and to other underground tree taxa. Firstly, it was expected that within a genus underground trees occupy a more extreme niche than their open- and closed-ecosystem congeners. Secondly, we hypothesized that underground trees of different genera inhabit distinct niches exposed to varying extremes of different environmental pressures. Our approach provides a novel quantification of multiple underground tree niches, comparing overlaps among congeners and genera to unearth the environments underground trees inhabit.

## MATERIALS AND METHODS

### Study area and taxa

The study area is the Afrotropics delimited as subcontinental mainland Africa south of the Sahara (Fig. 1A). Current use of the term ‘geoxyle’ extends to a morphological diversity of suffrutescent subshrubs that resprout from woody underground structures, including woody rhizomes, xylopodia or lignotubers (Lamont *et al*., 2017; Pausas *et al.*, 2018; Meller *et al.*, 2022), but reflects taxa that also have herbaceous congeners. We comply with definitions of underground trees as a subset of geoxyles that have woody tree, shrub or liana congeneric relatives (White, 1976; Simon *et al.*, 2009; Maurin *et al.*, 2014).

To compare the biogeography of underground trees and congeners, four woody genera that are widespread and well studied were selected from the list of African underground tree species by Maurin *et al.* (2014): *Parinari* (Chrysobalanaceae), *Ozoroa* (Anacardiaceae), *Syzygium* (Myrtaceae) and *Lannea* (Anacardiaceae). Except for *Syzygium*, these genera include more than one accepted obligate underground tree taxon (Table 1). Based on past ecological research and distribution records, it was understood that underground trees in these genera span the rainfall gradient of open ecosystems in the Afrotropics and have potential associations with different environmental controls (Table 1).

**Table 1.** Defined habit/habitat groups and counts per genus of taxa and occurrence records after cleaning. Hypothesized disturbances influencing the distribution of underground trees per genus are included based on the literature for *Parinari* (Zigelski *et al*., 2019*a*; Finckh *et al*., 2021; Gomes *et al.*, 2021*a*), *Ozoroa* (Revermann *et al.*, 2017), *Syzygium* (White, 1976; Maurin *et al.*, 2014; Zigelski *et al*., 2018, 2019*a*, *b*) and *Lannea* (Chidumayo, 2006, 2019). The list of all taxa used in analysis is available (https://doi.org/10.5281/zenodo.7031843).

Genus	Hypothesized disturbance	Habit/habitat group							
		Underground tree (UT)		Open-ecosystem congener (OE)		Closed-ecosystem congener (CE)		Total	
		A subshrub with expansive woody organs and congeneric tree relatives growing below ground in an open-canopy savanna or grassland ecosystem		A tree or shrub growing above ground in an open-canopy savanna or grassland ecosystem		A tree or shrub growing above ground in a closed-canopy or forest ecosystem			
		Taxa	Records	Taxa	Records	Taxa	Records	Taxa	Records
*Parinari* (Chrysobalanaceae)	Fire, herbivory, frost, waterlogging, soil properties	3	44	1	2912	4	404	8	3360
*Ozoroa* (Anacardiaceae)	Fire, herbivory, frost, soil properties	8	56	26	482	NA	NA	34	538
*Syzygium* (Myrtaceae)	Waterlogging, soil properties	1	38	5	394	29	1210	35	1642
*Lannea* (Anacardiaceae)	Fire, herbivory	5	64	22	2538	5	2916	32	5518
**Total**		17	202	54	6326	38	4530	**109**	**11058**

The underground tree *Parinari capensis* dominates many sandy plains and suffrutex grasslands (Zigelski *et al*., 2019*a*) or wetland edges (Coates-Palgrave, 2002), where its distribution can be driven by frost above the treeline (Finckh *et al*., 2021) as well as by fire and soil characteristics (Gomes *et al.*, 2021*a*). *P. capensis* and its two subspecies are compared in this study to the tree congener *P. curatellifolia*, which is abundant and widespread across southern African open ecosystems (Coates-Palgrave, 2002), and to four trees/shrubs in closed ecosystems (Table 1).

The genus *Ozoroa* includes an unusually high diversity of underground trees (17 taxa; Maurin *et al.*, 2014) of which eight were analysed here with 26 trees/shrubs (Table 1) that occur exclusively within Afrotropical open ecosystems (Coates-Palgrave, 2002). *Ozoroa* underground trees are prolific on hillslope grasslands of Kalahari sand (Revermann *et al.*, 2017).

In *Syzygium*, the only obligate underground tree, *Syzygium guineense* subsp. *huillense*, is restricted to the peripheries of seasonally inundated wetlands on sandy or clayey soils (White, 1976). *S. cordatum* and *S. guineense* subsp. *macrocarpum* sometimes adopt the suffrutescent habit in stressful conditions (White, 1976; Zigelski *et al*., 2018) but are usually tall trees (Coates-Palgrave, 2002) and are therefore considered tree congeners in this study since sampling is most likely to recognize them as such. Here, *S. guineense* subsp. *huillense* is examined relative to five open-ecosystem trees/shrubs and 29 closed-ecosystem trees/shrubs (Table 1). *Syzygium* has origins in wet biomes (Zigelski *et al.*, 2019*b*) and its trees/shrubs inhabit mesic forests, interfluves, riverbanks or refugia with ample rainfall/groundwater (Coates-Palgrave, 2002).

The underground tree *Lannea edulis* is especially well recognized as being adapted to fire in its morphology, phenology and population ecology (Chidumayo, 2019). Here, five *Lannea* underground trees are compared with 22 open-ecosystem trees/shrubs and five closed-ecosystem trees/shrubs. *Lannea* trees also grow a below-ground rootstock (Coates-Palgrave, 2002), while its shrubs are a common ground layer taxa in drier miombo (Chidumayo, 1987).

### Attribution of habit and habitat

A genus-level approach was used to compare the environmental niche of underground trees with their congeners, to both control for evolution and minimize the high potential for error in identification at species level (cf. Goodwin *et al.*, 2015) especially in tropical groups. Therefore, within a genus, each taxon (species, subspecies or variety) was assigned to one of three composite categories representing habit and habitat as ‘underground tree’, ‘open-ecosystem congener’ (OE congener) or ‘closed-ecosystem congener’ (CE congener). Underground trees occur in open grassy ecosystems only (White, 1976; Maurin *et al.*, 2014; Meller *et al.*, 2022). Hence, the distinction between open and closed ecosystems among congeners was considered to represent distinct eco-evolutionary environmental controls as open and closed ecosystems are shaped by different drivers and processes related to disturbance, ground layer light availability and microclimates (Oliveras and Malhi, 2016; Bond, 2019; Keith *et al.*, 2022). Categorization into habit/habitat groups (Table 1) was based on descriptions of taxa and the ecosystems with which they are associated available in *Plants of the World Online* (POWO, 2022) and online flora accounts such as *Flora Zambesiaca* (Exell and Wild, 1960) and the African Plants Database (Version 4.0.0). Descriptors associated with open ecosystems included: ‘grassland’, ‘wooded grassland’, ‘savanna’, ‘deciduous bushlands’, ‘open forest’, ‘wetland’ and ‘vlei’ (marshy depression). Terms to describe closed ecosystems included: ‘evergreen forest’, ‘montane forest’, ‘riparian forest’, ‘rain forest’ and ‘secondary forest’. The final list of taxa used in this analysis and associated habit/habitat groups is available (https://doi.org/10.5281/zenodo.7031843).

### Occurrence data

Occurrence records were sourced through the Global Biodiversity Information Facility (GBIF; https://doi.org/10.15468/dd.jh5csv). Subsequent data analysis used R 4.1.1 (R Core Team, 2021). All coordinate points of occurrence for accepted and georeferenced taxa in the four genera were downloaded through the R package ‘rgbif’ (Chamberlain *et al.*, 2022). Subspecies and varieties were preserved in the data and synonyms were merged to nomenclature accepted in the *Synonymic Checklists of Vascular Plants of the World* (Hassler, 2022). To clean data, the R package ‘CoordinateCleaner’ (Zizka *et al.*, 2019) was used to remove duplicate records and those located in the sea, on country centroids or at biodiversity institutions. Further data cleaning omitted records located on islands, including Madagascar. A resulting total of 11 058 records were obtained for 109 taxa in the four study genera (Table 1). Points of occurrence are presented in Supplementary Data Figs S1–S4, with the sampling density based on the number of all records per quarter-degree grid square in Supplementary Data Fig. S5.

### Environmental variables

Nine environmental variables spanning climate, seasonality, disturbance and hydrology were compiled to define the environmental space occupied by each habit/habitat group. The datasets and justifications for their use are outlined in Table 2. Each parameter was selected *a priori* as an environmental factor understood to be an eco-evolutionary driver of Afrotropical vegetation and underground tree dynamics across spatiotemporal scales (Fig. 1; Table 1). Particular attention was given to representing herbivory and waterlogging, which have been excluded from prior analyses but proposed as correlates of underground tree distributions (White, 1976; Maurin *et al*., 2014; Meller *et al*., 2022). In this context, herbivory can be considered an indirect process maintaining open ecosystems that underground trees benefit from rather than directly representing consumption of the underground trees themselves. A dataset for the topographic wetness index (TWI) was generated as a proxy for waterlogging (Supplementary Data Method S1 and Supplementary Data Fig. S6). A correlation matrix confirmed that there is no autocorrelation between standardized values of the environmental parameters selected (Supplementary Data Table S1).

**Table 2.** Nine environmental variables were used to assess the niche of underground trees and their open- and closed-ecosystem congeners.

Sort	Scale of influence	Variable	Code	Source	Justification
Climate	Regional	Mean annual air temperature – bio1 (°C)	MAT	Karger *et al.* (2017)	Temperature and temperature seasonality are known to be broadly ecologically relevant to vegetation variation along latitudinal and altitudinal gradients (De Frenne *et al.*, 2013).
		Temperature seasonality – bio4 (°C)	TS		
		Annual precipitation amount – bio12 (mm)	AP		Precipitation and precipitation seasonality have been shown to be key to structuring seasonally dry ecosystems and their limits across the global tropics (Lehmann *et al.*, 2011). These dimensions of the environment combine to determine climatic growing conditions and are strong drivers of plant productivity gradients.
		Precipitation seasonality – bio15 (%) (coefficient of variation)	PS		
		Mean monthly precipitation amount of the driest quarter – bio17 (mm)	DP		
Stress and disturbance	Landscape and local	Mean annual frost days 1990–2019	Frost	Harris *et al.* (2020)	Frost is an attribute of climate that causes thermal stress to plants (Finckh *et al*., 2016), increasingly recognized as an unexpectedly influential driver of tropical vegetation dynamics and underground trees in particular (Finckh *et al*., 2016, 2021; Meller *et al.*, 2022).
		Burned area (km^2^)	Fire	Phelps *et al.* (2022) derived from Giglio *et al.* (2018)	Fire is considered the common explanatory control on underground tree evolution (White, 1976; Maurin *et al.*, 2014; Davies *et al.*, 2016; Lamont *et al*., 2017).
		Herbivore biomass (kg/km^2^)	Hrbv	Hempson *et al*. (2015)	Mammal herbivory has comparable but contrasting effects to fire (Archibald and Hempson, 2016) as an above-ground consumer of vegetation that can limit woody plant growth and maintains open ecosystems (Bond, 2005). Although recognized for potential evolutionary influence on underground trees (Maurin *et al.*, 2014; Meller *et al.*, 2022), herbivory had not yet been incorporated into analyses. The dataset produced by Hempson *et al*. (2015) is derived from models of herbivore censuses and habitat preferences, excluding elephants so as not to overestimate biomass or mask patterns of other species.
		Topographic wetness index (TWI)	TWI	Derived from SRTM (2013)	TWI is a proxy for soil moisture that can be derived from digital elevation models (DEMs). Previously, TWI has been linked to patterns of plant species richness (Sørensen et al., 2006) and biomass (Xu et al., 2015). TWI also represents the influence of topography, with catenary variation (Sørensen *et al.*, 2006) and other soil properties significant to plants.

### Data extraction

From the nine datasets (Table 2), environmental values were obtained for each georeferenced GBIF occurrence record per study taxon. Additionally, a data sample was created from 100 000 random points across the Afrotropics. To extract values, the R package ‘raster’ (Hijmans *et al.*, 2015) was used to stack the data layers under the same projection and mask them to the Afrotropics using the Terrestrial Ecoregions of the World (Olson *et al.*, 2001). Records with ‘Not Applicable’ (NA) values were omitted. The environmental values (https://doi.org/10.5281/zenodo.7031862) were subsequently grouped according to genus and habit/habitat group of the associated taxa (Table 1). Thus, spectra of data were collated that distinguish environmental spaces inhabited by underground trees, OE and CE congeners of the study genera, as well as the background environment of the Afrotropics.

### Environmental space

To first evaluate similarity and dissimilarity in the environmental preferences of underground trees and congeners across genera, occurrence densities of taxa were plotted along gradients of nine environmental variables (Table 2). Raincloud plots visualize the distribution of raw occurrence data, means, standard deviations and errors alongside the probability of species occurrence (Allen *et al.*, 2021). These were produced using the R package ‘ggplot2’ (Wickham, 2016) per genus and habit/habitat group (Table 1). For statistical analysis, values of environmental variables were transformed (either by square root or log) to normal distributions if required. A one-way ANOVA then tested for significant differences among means.

Second, associations of habit/habitat groups and genera with environmental variables in multidimensional space were evaluated through a principal component analysis (PCA). Based on covariance, relationships are demonstrated between variables and their relative contributions to the distribution of data. Environmental values were standardized to a mean of 0 and the absence of autocorrelation between variables was re-confirmed. In the PCA, the random data sample from 100 000 locales represents a background of the wider Afrotropical environment within which occurrences of each group are situated. Biplots for the first and second components were produced, demonstrating the contribution of variables to explanatory power. The position of groups in the PCA, with ellipses at the 95 % confidence interval, comprehensively indicates the environmental contexts across which different taxa occur.

### Niche overlap and similarity

Direct comparisons of species–environment relationships were made for pairwise taxa combinations grouped by genus and habit/habitat (Table 1), and using spatial data layers of the nine selected environmental variables (Table 2) that cover the Afrotropical study area. The environmental niche overlap was calculated in the R package ‘ecospat’ (Di Cola *et al.*, 2017), which implements the PCA–environment ordination method outlined by Broennimann *et al.* (2012). Estimates of niche overlap between taxa are based on the *D* (Schoener, 1970) and *I* (Warren *et al.*, 2021) metrics, ranging from values of 0 (no overlap) to 1 (entire overlap). To determine whether observed *D* and *I* statistics differed significantly from those expected by chance, a recommended 100 randomized model iterations were compared within the background Afrotropical environment using methodologies by Warren *et al*. (2008). Statistically significant values are indicative that two taxa, or in this case taxa groups, occupy dissimilar environmental niche spaces.

### Geographical range

Occurrence records were used to calculate estimates of range size for each underground tree, OE and CE congener of *Parinari, Ozoroa, Syzygium* and *Lannea* in the Afrotropics. When available data are presence-only, as here, α hulls drawn between points of occurrence represent a species’ range size as its extent of occurrence (EOO) (IUCN, 1994). An α of 200 km with a 10-km buffer was selected based on the findings of Mashau *et al.* (2021) where, in African open ecosystems, this value appropriately reconciled over- and underestimations across regions with scarcity and disparity in sampling density (Supplementary Data Fig. S5). To calculate EOO per taxon, the function ‘EOO computing’ was used in the R package ‘ConR’ (Dauby *et al.*, 2017). The EOO calculation requires a minimum of three unique occurrences, and therefore taxa with fewer records were not included. Maps were produced in QGIS (Version 3.16.9) to spatially represent range size geometries and geographical overlap/separation among habit/habitat groups, which point occurrence maps were too dense to demonstrate (Supplementary Data Figs S1–S5). After logging EOO values, a one-way ANOVA tested for significant difference in range size among habit/habitat and genus.

## RESULTS

### Biogeography of underground trees and congeners

**Fig. 2.**
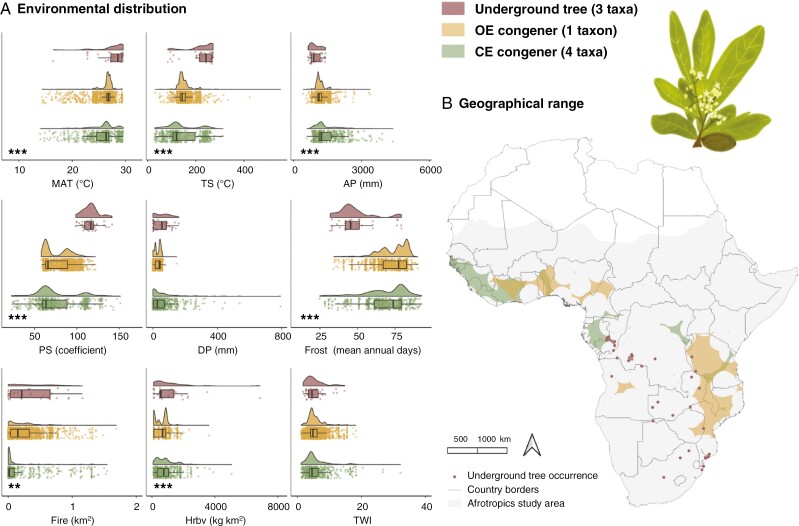
Environment and geography of *Parinari* (Chrysobalanaceae) taxa as underground trees, open-ecosystem (OE) congeners or closed-ecosystem (CE) congeners in the Afrotropics. (A) Environmental distribution of *Parinari* as a density of occurrence along environmental gradients of mean annual temperature (MAT), temperature seasonality (TS), annual precipitation (AP), precipitation seasonality (PS), dry season precipitation (DP), frost, fire, herbivory (Hrbv) and the topographic wetness index (TWI). Statistical significance of a one-way ANVOA is presented as very highly significant (***P < 0.001) and highly significant (**P < 0.01). (B) Geographical range sizes of *Parinari* taxa calculated (Supplementary Data Fig. S7) and mapped as extents of occurrence. Diagram of the underground tree *P. capensis* subsp. *capensis*.

**Fig. 3.**
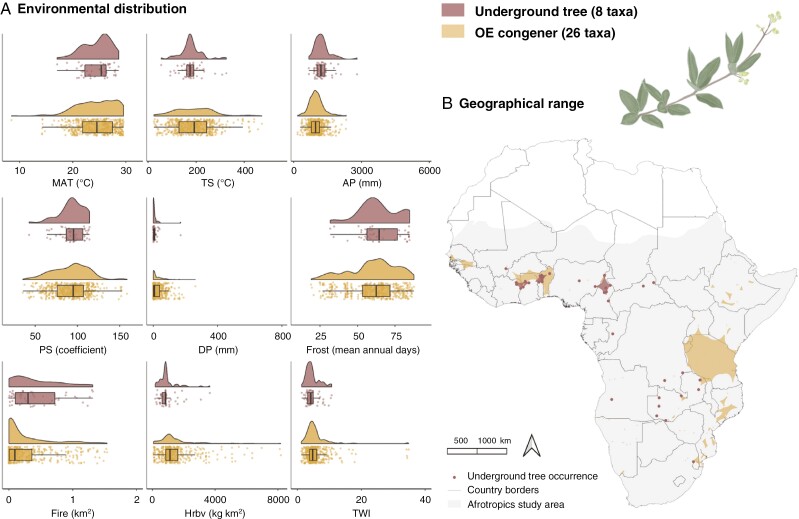
Environment and geography of *Ozoroa* (Anacardiaceae) taxa as underground trees or open-ecosystem (OE) congeners in the Afrotropics. (A) Environmental distribution of *Ozoroa* as a density of occurrence along environmental gradients of mean annual temperature (MAT), temperature seasonality (TS), annual precipitation (AP), precipitation seasonality (PS), dry season precipitation (DP), frost, fire, herbivory (Hrbv) and the topographic wetness index (TWI). Statistical significance of a one-way ANVOA is presented as very highly significant (***P < 0.001), highly significant (**P < 0.01) and significant (*P < 0.05). (B) Geographical range sizes of *Ozoroa* taxa calculated (Supplementary Data Fig. S7) and mapped as extents of occurrence. Diagram of the underground tree *O. homblei*.

**Fig. 4.**
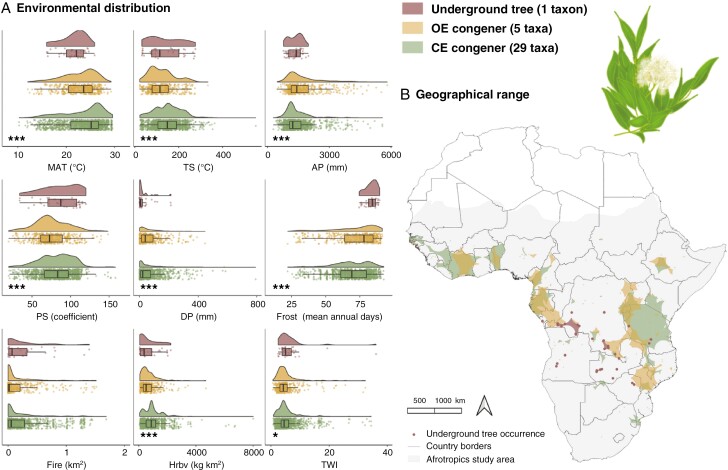
Environment and geography of *Syzygium* (Myrtaceae) taxa as underground trees, open-ecosystem (OE) congeners or closed-ecosystem (CE) congeners in the Afrotropics. (A) Environmental distribution of *Syzygium* as a density of occurrence along environmental gradients of mean annual temperature (MAT), temperature seasonality (TS), annual precipitation (AP), precipitation seasonality (PS), dry season precipitation (DP), frost, fire, herbivory (Hrbv) and the topographic wetness index (TWI). Statistical significance of a one-way ANVOA is presented as very highly significant (***P < 0.001). (B) Geographical range sizes of *Syzygium* taxa calculated (Supplementary Data Fig. S7) and mapped as extents of occurrence. Diagram of the underground tree *S. guineense* subsp. *huillense*.

**Fig. 5.**
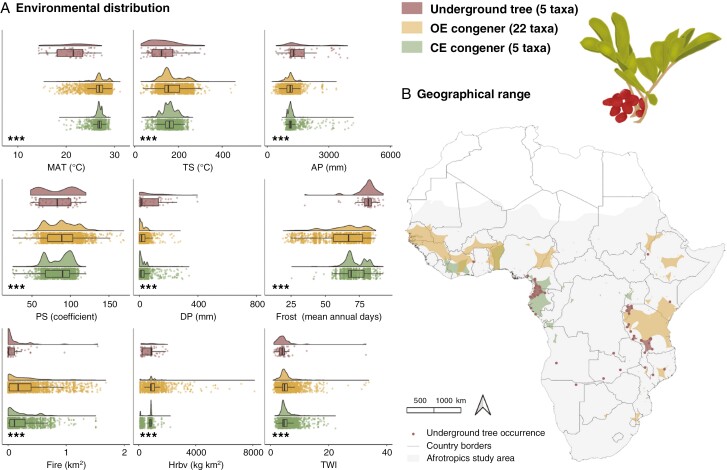
Environment and geography of *Lannea* (Anacardiaceae) taxa as underground trees, open-ecosystem (OE) congeners or closed-ecosystem (CE) congeners in the Afrotropics. (A) Environmental distribution of *Lannea* as a density of occurrence along environmental gradients of mean annual temperature (MAT), temperature seasonality (TS), annual precipitation (AP), precipitation seasonality (PS), dry season precipitation (DP), frost, fire, herbivory (Hrbv) and the topographic wetness index (TWI). Statistical significance of a one-way ANVOA is presented as very highly significant (***P < 0.001) and significant (*P < 0.05). (B) Geographical range sizes of *Lannea* taxa calculated (Supplementary Data Fig. S7) and mapped as extents of occurrence. Diagram of the underground tree *Lannea edulis.*

Characterizing the composite habit/habitat groups within each genus, Figs 2–5 present the distributions of taxa occurrence along environmental gradients and alongside range size maps. Reporting of values focuses on frost, fire, herbivory and TWI, given their particular eco-evolutionary significance for underground trees (Fig. 1), and the genera are ordered from the most arid (*Parinari*) to the most mesic (*Lannea*) conditions inhabited by underground trees based on annual precipitation. Values of overall environmental preferences for each habit/habitat group per genus are summarized in Supplementary Data Table S2.

#### Parinari.

In arid conditions (Fig. 2A), *Parinari* underground trees occur among high mean temperatures (27.57 ± 2.74 °C) and low annual precipitation (952.75 ± 222.00 mm) conditions with significant differences from the overall congener means (25.79 ± 2.61 °C and 1369.52 ± 437.65 mm). There is also a greater seasonal variation in these parameters but low and insignificant differences in dry season precipitation. Frost is significantly associated with *Parinari* underground trees with a mean frost frequency of 48.57 (± 13.53) d, which is 33.36 % lower than the OE congener mean (72.88 ± 9.28) and 22.39 % lower than the CE congener mean (62.59 ± 14.82). Contrastingly, under the hot and dry conditions, fire activity is significantly greater where *Parinari* underground trees occur, with a mean burned area of 0.33 (± 0.31) km^2^, 35.18 % greater than for OE congeners (0.22 ± 0.24 km^2^) and 27.8 % greater than for CE congeners (0.24 ± 0.40 km^2^). A mean herbivory value of 1,053.79 kg km^−2^ also signifies significantly greater herbivory pressure in the underground tree environment (*P *< 0.001) by 43.6 and 23.52 % compared, respectively, with OE congeners (593.94 ± 433.33 kg km^−2^) and CE congeners (805.94 ± 629.91 kg km^−2^). Soil moisture is similar in environments of *Parinari* underground trees and congeners. The underground tree TWI mean is 5.31 (± 2.98), just 0.54 % greater than the OE congener mean (5.29 ± 2.36) but 15.15 % lower than the CE congener mean (6.26 ± 4.60). Further, *Parinari* underground trees have separate geographies and smaller range sizes than congeners (Fig. 2B) with average range size of 14 275 km^2^ compared with the overall genus mean of 699 014 km^2^.

#### Ozoroa.

*Ozoroa* underground trees generally inhabit similarly warm areas (with a mean of 24.50 °C) to congeners that occur only in comparable open ecosystems (Fig. 3A), but with a significant greater mean annual precipitation (1257.10 mm) than congeners (1022.67 mm). Dry season precipitation is also significantly greater for underground trees, despite insignificant differences in seasonality. Where underground trees occur at a mean of 64.31 (± 13.49) frost days, frost frequency is just 6.17 % greater, but significant, than for OE congeners (60.34 ± 16.50 frost days). In contrast, fire activity is 32.87 % greater for underground trees at a mean of 0.43 (± 0.39) km^2^ than OE congeners (0.29 ± 0.38 km^2^), but the difference is insignificant. Of the stresses, therefore, most dissimilarity between *Ozoroa* underground trees and OE congeners is evidenced by herbivory, for which the mean is significantly less by 3.94 % in the underground tree (945.77 ± 628.74 kg km^−2^) than OE congener (1431.796 ± 1163.47 kg km^−2^) environments. The mean TWI value is also significantly less, by 15 %, where *Ozoroa* underground trees occur (4.66 ± 2.12) than for OE congeners (5.52 ± 3.68). The mean range size of *Ozoroa* underground trees is 24 860 km^2^ compared with the genus mean of 34 286 km^2^ and little overlap with OE congeners is shown within this more restricted geographical range (Fig. 3B).

#### Syzygium.

Cooler, wetter (in precipitation and soil moisture) and more seasonal conditions characterize the underground tree environment of *S. guineense* subsp. *huillense* compared with its congeners (Fig. 4A). The mean temperature where underground trees occur (21.42 ± 3.50 °C) is less than for OE and CE congeners (22.91 ± 3.94 °C), significant annually and seasonally. *Syzygium* underground trees receive more mean precipitation (1339.24 mm) than congeners (1555.12 mm), also significant annually and seasonally. Stress by frost is significantly greater for underground trees, with a mean of 83.19 (± 4.02) frost days, which is more frequent by 13.8 % than for OE congeners (71.69 ± 16.34) and by 18.02 % than for CE congeners (68.20 ± 13.03). Fire is demonstrably greater, by 28.4 %, where underground trees occur, with a mean of 0.26 (± 0.36) km^2^ than for OE congeners (0.18 ± 0.32 km^2^) but greater by 5.95 % compared with CE congeners (0.24 ± 0.35 km^2^). Burned area is the only environmental parameter between which there is no statistically significant difference in mean values among *Syzygium* habit/habitat groups. Pressure by herbivory, however, is lower in the underground tree environment, with a mean of 644.37 (± 675.35) kg km^−2^. This is significantly less than OE congener (688.95 ± 567.88 kg km^−2^) and CE congener (977.60 ± 736.62 kg km^−2^) herbivory means. A high mean TWI value for *Syzygium* underground trees (6.43 ± 5.83) indicates lower positioning on a hillslope, further down a catena profile where runoff accumulates and soils become saturated. The underground tree TWI value is significantly greater than the OE congener mean (4.94 ± 3.74) by 23.15 % and the CE congener mean (5.46 ± 4.12) by 15.08 %. The mean underground tree range size is 45 121 km^2^ compared with a genus mean of 210 341 km^2^, making *Syzygium* taxa the most widely distributed taxa overall in this study (Fig. 4B).

#### Lannea.

Of the underground trees studied*, Lannea* taxa inhabit the most mesic environments (Fig. 5A). For all environmental parameters, there is a high significant difference between mean values at the occurrences of underground trees, OE and CE congeners, emphasizing their occupation of unique environmental spaces. *Lannea* underground trees occur with lower mean temperature (20.77 ± 3.31 °C), higher precipitation both annually (1591 ± 838.83 mm) and in the dry season, and less overall seasonality relative to both OE and CE congeners (with means of 26.73 ± 1.49 °C and 1131.87 ± 260.66 mm). *Lannea* underground trees are exposed to more frost, experiencing 80.45 (± 8.6) mean annual frost days, which is 21 and 11 % greater than values for OE (63.0 ± 14.5) and CE (70.8 ± 7.1) congeners, respectively. Cooler and wetter conditions align with environments associated with a mean burned area of 0.11 (± 0.25) km^2^ compared with the average burned area for OE congeners (0.31 ± 0.33 km^2^), which is 63 % less. Notably, 50 % less fire activity is experienced in the underground tree than in the CE congener (0.23 ± 0.24 km^2^) environments. *Lannea* underground trees are associated with a mean herbivory value of 746.55 (± 483.95) kg km^−2^, which is lower than the value for their OE congeners (958.37 ± 632.04 kg km^−2^) by 22.10 %, but just 3.80 % more than for CE congeners (718.15 ± 296.99 kg km^−2^). The mean TWI for *Lannea* underground trees is 4.29 (± 4.02), compared with greater means by 20 % for OE congeners (5.37 ± 2.82) and 17.66 % for CE congeners (5.21 ± 2.12). Although these underground trees receive more precipitation, soil moisture is lower since runoff does not accumulate upslope, where TWI values are lower. *Lannea* underground trees are distributed with a smaller range size of 25 593 km^2^ compared to the genus mean of 106 247 km^2^ and demonstrate little spatial overlap with congeners (Fig. 5B).

### Environmental distinctiveness

**Fig. 6.**
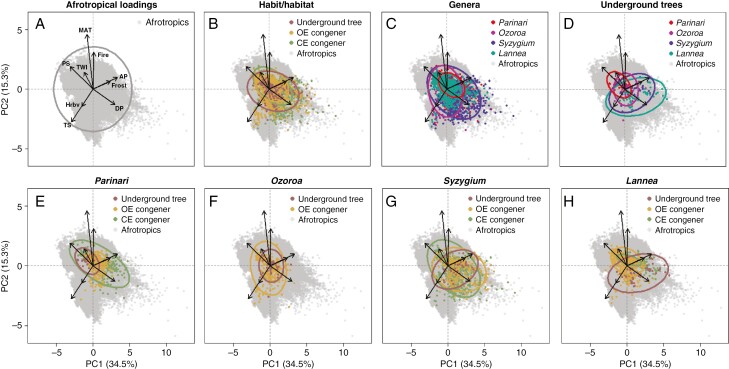
Principal component analyses show the multidimensional space for environmental variables of mean annual temperature (MAT), temperature seasonality (TS), annual precipitation (AP), precipitation seasonality (PS), dry season precipitation (DP), frost, fire, herbivory (Hrbv) and the topographic wetness index (TWI), corresponding to Table 2 and Figs 2–5. Relationships among variables are shown for all taxa in (A) the Afrotropical loadings. Overlap of 95 % confidence ellipses is shown for underground trees, open ecosystem (OE) and closed ecosystem (CE) congeners grouped by (B) habit/habitat according to Table 2, (C) all taxa by genus, (D) underground tree by genus, (E) habit/habitat in *Parinari* (Chrysobalanaceae), (F) habit/habitat in *Ozoroa* (Anacardiaceae), (G) habit/habitat in *Syzygium* (Myrtaceae) and (H) habit/habitat in *Lannea* (Anacardiaceae).

In multidimensional environmental space, the PCA determined relationships between variables in the Afrotropical environment and their relative influence in explaining the distribution of occurrence data (Fig. 6A). Based on eigenvalues, variance of data is sufficiently explained by the first four components (73.8 %), with the first two accounting for half (49.8%). Annual precipitation primarily drives the first component, where seasonality variables (precipitation seasonality, dry season precipitation and temperature seasonality, respectively) contribute similar explanatory power. Of the environmental stress variables, frost explains most variance, followed by herbivory, TWI then fire. However, in the second component, mostly driven by temperature, fire prevails among the stress variables.

Overall, underground trees as a habit/habitat group demonstrate a broader and less restricted distribution across environmental spectra than their congener, to include greater extremes of bioclimatic, seasonality and disturbance variables (Fig. 6B). Grouping all taxa by genus also evidences niche separation across the PCA (Fig. 6C). Hence, distinguishing underground trees by genus (Fig. 6D) reveals low to intermediate overlap (Table 3). There is no statistically significant dissimilarity by genus between any pair of underground trees, however, due to observed overlaps in the PCA and relatively small sample sizes.

**Table 3.**
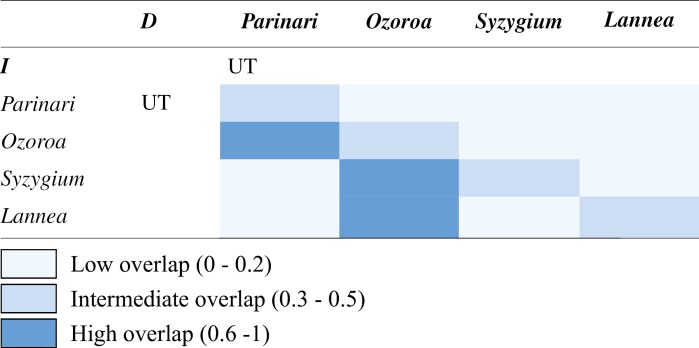
Matrices of niche overlap values of *D* and *I* (Broennimann *et al.*, 2012) based on overlap in a PCA among underground trees (UT) per genus.

Grouping taxa by both genus and habit/habitat further separates niches along environmental preferences (Fig. 6E–H). The underground tree niche consistently demonstrates low overlap to the niche of congeners (Table 4), with low *D* values (0.05–0.39) to intermediate *I* values (0.19–0.61) (Broennimann *et al.*, 2012). Statistically significant (*P* < 0.05) dissimilarity is seen between *Ozoroa* underground trees and its OE congeners, despite the high *I* value (Table 4).

**Table 4.**
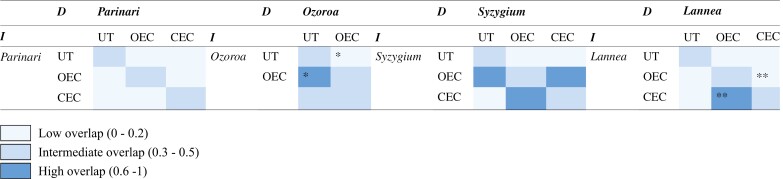
Matrices of niche overlap values of *D* and *I* (Broennimann *et al.*, 2012) based on overlap in a PCA among all habit/habitat groups [underground tree (UT), open ecosystem congener (OEC) and closed ecosystem congener (CEC)] for each genus. Asterisks indicate a highly significant (**P < 0.01) or significant (*P < 0.05) difference in the niche.

*Parinari* and *Ozoroa* underground trees occupy narrower niches, also within the environmental range of their congeners. These genera overall represent arid, hotter and drier environments with more precipitation seasonality that are more aligned with fire activity than frost (Fig. 6E, F). *Parinari* and *Ozoroa* underground trees again demonstrate low to intermediate overlap but not significantly different niches (*D* = 0.33, *I* = 0.51, *P* > 0.05). Comparatively, *Syzygium* and *Lannea* represent broad underground tree niches, spanning more extremes across the PCA surface whereby seasonality distinguishes the alignment of underground trees from congeners towards inhabiting more mesic, cooler, wetter frost-prone environments (Fig. 6G, H). *Syzygium* and *Lannea* underground trees demonstrate low overlap but are not significantly distinct (*D *= 0.22, *I* = 0.44, *P* > 0.05).

### Range size

Separate distributions of underground trees and congeners among genera are suggested in geographical space. Range sizes are on average 15.29 % (± 9.67 %) smaller for underground trees than OE congeners and 20.89 % (± 10.50 %) smaller than CE congeners but without statistical differences in the mean logged values (Supplementary Data Fig. S7a). Range sizes among taxa, of all habit/habitat groups, are significantly distinguishable by genus (*P* < 0.05) (Supplementary Data Fig. S7a). *Ozoroa* demonstrates the smallest mean range size, in line with the exclusive occurrence of its taxa in open ecosystems.

## DISCUSSION

### Do underground trees inhabit a distinct niche relative to congeners?

Across Afrotropical environments, underground trees occupy more extreme environments and stressed niches than their tall tree/shrub congeners. When underground trees are considered as a single group incorporating the four genera, the environmental niche occupied is broader than that of OE and CE congeners. Hence, grouping all underground tree taxa masks how their niche stretches into diverse extreme environments and demonstrates the necessity of understanding the variety of geographical contexts in which underground trees are found.

By demonstrating distinct niches among life forms, underground trees can be considered an advantageous strategy to survive diverse and potentially multiple disturbances. Woody plant recruitment from juvenile to adult size classes is typically limited by above-ground cycles of top-kill through ‘fire traps’ (Wakeling *et al*., 2011; Holdo *et al*., 2014), ‘freeze traps’ (Whitecross *et al*., 2012; Hoffmann *et al.*, 2019) or ‘browse traps’ (Staver *et al.*, 2009; Staver and Bond, 2014). We also propose a ‘waterlogging trap’ mediated by below-ground edaphic factors, based on the relevance of TWI in this study and further supported by evidence that seasonal inundation limits tree cover (Daskin *et al*., 2019) and rates of shoot extension in juvenile tropical trees (Parolin *et al.*, 2016). Seasonal inundation creates unfavourable anoxic soil conditions at a time when tall trees would need to invest greatly in vertical growth to escape the fire trap especially, while underground trees can remain dormant below ground. Some non-obligate underground trees known to be confined to wetland edges, including *S. cordatum* and *S. guineense* subsp. *macrocarpum*, can outgrow the suffrutescent life form when protected from environmental stress (White, 1976; Zigelski *et al*., 2019*a*). As opposed to favouring recruitment through disturbance zones, underground trees demonstrate life history strategies for a persistence niche (Bond and Midgley, 2001) including *in situ* resprouting, clonal growth and heterochrony that allows flowering and fruiting below disturbance zones (Li and Johnston, 2000; Maurin *et al.*, 2014). The suffrutescent life form could therefore be the most viable option for a woody plant to endure different and interacting environmental extremes of fire, frost, herbivory and waterlogging.

Although underground trees and woody congeners occupy different environmental spaces and low overlaps demonstrate separation in their niches (Sankaran *et al*., 2004), they were not found to be entirely dissimilar overall. Potentially, discrepancies between overlap and dissimilarity are due to the necessity to analyse datasets that do not represent more local heterogeneity. Yet, interestingly, the underground tree niche is more dissimilar to the environmental space inhabited by congeners in open ecosystems than closed ecosystems. Competitive exclusion can explain the absence of underground trees in forests despite their origins in some forest lineages (Maurin *et al.*, 2014) where tall trees limit root space and light availability to the ground layer (Pilon *et al.*, 2021), unrestricted by demographic bottlenecks resulting from disturbance ‘traps’ (Holdo *et al*., 2014; Bond, 2019). Savannas generally represent a biogeographical paradox to competitive exclusion, with a species-rich herbaceous ground layer co-existing under varying tree cover and competing for the same limiting resources (Colgan and Asner, 2014). Where light competition is high, niche separation requires disturbances such as fire and herbivory (Scheiter and Higgins, 2007). Even minor differences in environmental niches can balance competitive exclusion (Colgan and Asner, 2014). Therefore, underground trees may have evolved in stressful environments because there is available and unshaded niche space where tall trees are filtered out.

Below-ground woody structures fulfil a range of functions beyond resprouting (Pausas *et al.*, 2018) and enable resource acquisition. Despite architectural and allometric differences, there is morphological resemblance between underground trees and their congeners, demonstrated by no substantial phenetic differences among reproductive traits of leaf, fruit and flower size (Meerts, 2017). While a low stature may see shade-intolerant underground trees overtopped in grassy ecosystems that mature in the wet season (White, 1976; Meller *et al.*, 2022), observations that they shift their vegetative phenology to leaf before or at the onset of the wet season likely facilitate co-existence with grasses (Zigelski *et al*., 2019*a*). Phenological niche separation whereby extensive below-ground structures enable banking of resources over a wet season is conceptually similar to the well-documented ‘pre-rain green up’ phenomenon of the Afrotropics (Archibald and Scholes, 2007). It could therefore be that adoption of a short stature by relocation of woody biomass below ground, which is speculated to be simple developmentally (Simon *et al.*, 2009) and genetically (Simon and Pennington, 2012), is a sufficient strategy for underground trees to colonize a diversity of stress-prone niches as we observe. Phenological observations of underground trees (e.g. Chidumayo, 2019) are required to determine whether the diversity of Afrotropical underground trees share similar phenologies across the range of environmental extremes such as in heavily grazed (e.g. *Parinari*) versus seasonally flooded (e.g. *Syzygium*) environments.

### Do underground trees inhabit distinct niches among genera?

Underground trees of the four study genera demonstrate contrasting environmental distributions, uncovering niche- and taxonomic-specific preferences. With precipitation primarily explaining environmental distributions of the study genera in the PCA (see also Lehmann *et al.*, 2011), the niche of underground trees in each genus is also associated with different disturbances along the rainfall gradient.

*Parinari* occupies the hottest and driest underground tree niche examined. The underground tree *P. capensis* has functional traits better adapted to water stress in sun-exposed open ecosystems relative to congeneric trees preferring mesic microclimates (Gomes *et al.*, 2019), confirmed by some of the highest rainfall values evidenced here for *Parinari* CE congeners. *P. capensis* is known to dominate dry suffrutex grasslands, such as in the Angolan Plateau (Revermann *et al.*, 2017) on low-nutrient sandy arenosols (Revermann and Finckh, 2013). Fire activity is high in the *Parinari* underground tree niche, and *Parinari* grasslands are particularly fire-prone, burning two out of every three years and almost twice as often as those dominated by *Brachystegia* underground trees (Gomes *et al.*, 2021*a*). Patterns of disturbance by fire and herbivory vary spatiotemporally, whereby herbivory peaks in generally lower rainfall environments than fire (Archibald and Hempson, 2016; Charles-Dominique *et al.*, 2016), shown here for the dry *Parinari* underground tree environment. While associated with high herbivory, *Parinari* underground trees (and others) are known to be relatively unpalatable. It may therefore be that direct changes to the underground tree habitat by selective grazing of grasses has indirect impacts on the underground tree niche by shaping and maintaining open ecosystems, such as by reducing light competition. Disturbances of herbivory and fire are highly manipulated by human activity (Asner *et al.*, 2004; Archibald, 2016), and *Parinari* suffrutex grasslands are preferred over others for conversion to agriculture (Gomes *et al.*, 2021*a*) where disturbances are suppressed. Although *Parinari* underground trees occur with the least frequent frost among genera in this study, frost burns have been observed on *P. curatellifolia* at suffrutex grassland/forest ecotones (Finckh *et al*., 2016), evidencing that there is interplay of stress and disturbance.

Across the underground tree study taxa, those in *Ozoroa* inhabit the most fire-prone environments, occurring with generally high rainfall. These conditions are favoured by Maurin *et al.* (2014) in explaining the evolutionary emergence of underground trees, and support observations by White (1976) that they occur in higher rainfall open ecosystems with frequent fires. Selecting for disturbance-adapted taxa, fire is fuelled by high grass productivity linked to high precipitation (Bond, 2008; Lehmann *et al.*, 2011). Underground trees can be deemed an indicator of African fire-maintained savannas and grasslands where forests could otherwise prevail (Maurin *et al.*, 2014 and references therein). Relative to their congeners, *Ozoroa* underground trees experience double the fire activity but similarly intermediate frost frequencies. Hence, *Ozoroa* underground trees colonize fire-driven landscapes, which could be in highlands as indicated by low TWI values, but perhaps geographically beyond where the ‘freeze trap’ is most influential.

In cool and wet conditions across a broad environmental space, *Syzygium* underground trees occur under the greatest frost frequency and the highest topographic potential for waterlogging in this analysis. These patterns indicate that *Syzygium* underground trees may be characteristic of frost-prone valley depressions where dambos or waterlogged soils occur at the bottom of a catena profile (Brunner *et al.*, 2004). Frost nights are recurrent in valleys and depressions, such as in the Angolan Plateau, by which topographic variation allows cold air to accumulate (Revermann and Finckh, 2013). The weather conditions for frost arise mostly on the grasslands and peatlands in the lower slopes or valley bottoms rather than the forested hillsides (Finckh *et al*., 2016). It has been suggested that the presence of waterlogged peatlands and small streams in valley depressions can exacerbate the generation of cold air (Finckh *et al*., 2021). Waterlogging can halt tree growth, causing a positive feedback where fewer trees generate cooler air (e.g. Lehner *et al*., 2017) and make deeper, more treeless valleys more prone to frost (Finckh *et al*., 2021). Further, dambos comprise grassy vegetation that also experiences frequent burning, maintaining an open tree cover that would favour shade-intolerant underground trees (Maurin *et al.*, 2014). Like trees, underground trees generally evade fully waterlogged sites for the well-drained peripheral interfluves (White, 1976; Zigelski *et al*., 2018, 2019*a*). However, with wet biogeographical origins (Zigelski *et al.*, 2019*b*), the underground tree *S. guineenese* ssp. *huillense* is confined to dambo peripheries (White, 1976; Maurin *et al.*, 2014; Zigelski *et al.*, 2019*a*) and evidently, by the findings of this study, occurs with topographic potential for high soil moisture or waterlogging.

*Lannea* underground trees were shown to occupy the broadest environmental space of those examined. Occurring under the coolest and wettest conditions, frost stands out as a stress in their niche. Fire activity is less in the underground tree niche than congener niche, with a lower mean than other underground trees. However, *Lannea* demonstrated the greatest maximum burned area of all underground trees. Dependency of the underground tree *L. edulis* on fire has been well detailed, by which burning was observed by Chidumayo (2019) as the main cause of aerial shoot dieback (Chidumayo, 2006), although frost was rare at that study site with a low diurnal temperature range. Since *Lannea* occupies the largest mean range size of the study genera and consequently a wide environmental niche, it is possible that fire is an important driver at the higher rainfall end of its distribution, whereas frost is also important at its drier and colder limits. Hence, while top-kill by frost was found to be the predominant stress correlate in this study, the range of environments occupied by *Lannea* underground trees suggests ‘fire traps’ and ‘freeze traps’ can be prevalent across the distribution of a single species (Holdo, 2007; Whitecross *et al*., 2012; Finckh *et al*., 2016). Furthermore, frost has been shown to reinforce the effects of fire on tropical vegetation dynamics (Hoffmann *et al.*, 2019).

### There is no single eco-evolutionary driver for underground trees

Underground tree growth forms have evolved convergently and are morphologically similar but our analyses show that, rather than selection against a single environmental stressor, phylogenetically unrelated underground trees occupy distinct environments. Our study confirms fire as a prevalent driver (Maurin *et al.*, 2014; Lamont *et al*., 2017) and frost as another key environmental control (Finckh *et al*., 2016; Meller *et al.*, 2022) that was poorly accounted for previously. However, these drivers are not an either-or, need not be mutually exclusive (Chidumayo, 2019) and are not the only extremes shaping underground tree biogeography. Our findings may still underrepresent just how profoundly stressful the underground tree niche is within a local ecosystem, relative to the wider Afrotropics. Limited availability of high-resolution data for environmental stresses, particularly microclimate and edaphic conditions, hinders incorporation of local-scale variability in subcontinental or regional studies. For example, the best available 0.5° resolution of frost occurrence (Harris *et al.*, 2020) used here underestimates by a magnitude of 10–40 times the frequencies observed in Angola by Meller *et al.* (2022). Nonetheless, the potential identification of local-scale impacts is supported by our findings of low niche overlaps, suggesting taxa restriction to the ecologies of specific niches.

At a global scale, taxa in other disturbance-prone and extreme environments also exhibit convergent evolution. A prominent example is Mediterranean climate systems, such as South Africa’s fynbos and California’s chaparral, where shrubs and herbs converge on strategies to cope with fire, including development of lignotubers and serotinous seed dispersal (Onstein *et al.*, 2015; Pausas *et al.*, 2018). Another example is tropical alpine regions, where large rosettes are a response to frost and the floral composition overall is a result of recruitment by long-distance dispersal of adapted flora (Kandziora *et al.*, 2022). For underground trees, however, it is likely that the life form is an adaptive result of *in situ* radiations by locally available lineages, even from across a penetrable or unstable biome boundary (Pennington and Hughes, 2014).

With findings that underground trees in different genera occupy separate niches among extremes of fire, frost, herbivory, waterlogging and climate, it is important to account for this variability in the management of grassy ecosystems. Impacts of land transformations in open ecosystems are especially destructive where below-ground biomass is uprooted, (Buisson *et al.*, 2019, 2022), such as by ploughing for intensive agriculture or afforestation (Stevens *et al*., 2022), with potentially comparable impacts to deforestation of contiguous miombo savanna woodlands (e.g. Gomes *et al.*, 2021*b*). Direct threats to underground trees are compounded by suppression of intrinsic disturbance regimes (Buisson *et al.*, 2019; Stevens *et al.*, 2022), misconceptions that devalue non-forest vegetation (Parr *et al.*, 2014; Silveira *et al.*, 2021) and limited capacity to monitor or research below-ground and short-stature biomass (Siebert *et al*., 2019). Furthermore, active restoration of below-ground biomass is currently complicated, requiring translocation of underground storage organs that are easily damaged (Le Stradic *et al.,* 2016) with long establishment times (Veldman *et al.*, 2015). Therefore, management strategies should maintain and regulate ecological processes that disturbance-adapted floras depend upon, such as through prescribed burning and grazing management (Buisson *et al*., 2019), as well as better incorporate below-ground biomass, its distribution and diversity. To support this, further research could identify potential effects of anthropogenic changes on below-ground biomass, for example the reduction of landscape-scale fires across Africa (Phelps *et al.*, 2022), which would be vastly improved by the development of fine-scale datasets and detailed local studies to represent landscape variability. Increased recognition of below-ground complexity and its informed, context-specific management is crucial to the resilience of grassy ecosystems and constituent disturbance-adapted biodiversity.

## SUPPLEMENTARY DATA

Supplementary data are available at *Annals of Botany* online and consist of the following. Figure S1: map of cleaned GBIF occurrence records in the Afrotropical study region for *Parinari* (Chrysobalanaceae) underground trees, open-ecosystem (OE) congeners and closed-ecosystem (CE) congeners. Figure S2: map of cleaned GBIF occurrence records in the Afrotropical study region for *Ozoroa* (Anacardiaceae) underground trees, open-ecosystem (OE) congeners and closed-ecosystem (CE) congeners. Figure S3: map of cleaned GBIF occurrence records in the Afrotropical study region for *Syzygium* (Myrtaceae) underground trees, open-ecosystem (OE) congeners and closed-ecosystem (CE) congeners. Figure S4: map of cleaned GBIF occurrence records in the Afrotropical study region for *Lannea* (Anacardiaceae) underground trees, open-ecosystem (OE) congeners and closed-ecosystem (CE) congeners. Figure S5: map showing sampling density as the number of cleaned GBIF occurrence records for all georeferenced taxa in the genera *Parinari* (Chrysobalanaceae)*, Ozoroa* (Anacardiaceae), *Syzygium* (Myrtaceae) and *Lannea* (Anacardiaceae) per quarter-degree grid square across the Afrotropics. Figure S6: map of the topographic wetness index (TWI) spatial database created for Africa in this study. Figure S7: logged extent of occurrence (EOO) range size for (a) taxa of all genera as underground trees, open-ecosystem congeners (OE congener) and closed-ecosystem congeners (CE congener); and (b) all taxa by genus with significant results (*P < 0.05) from a one-way ANOVA. Method S1: process used to derive a topographic wetness index for the African continent. Table S1: correlation matrix confirming no autocorrelation (all values <0.7) among standardized bioclimatic and environmental stress variables used in this study: mean annual temperature (MAT), temperature seasonality (TS), annual precipitation (AP), precipitation seasonality (PS), dry season precipitation (DP), frost, fire, herbivory (Hrbv) and the topographic wetness index (TWI). Table S2: mean values and standard deviation for occurrence data of each habit/habitat category [underground tree, open-ecosystem (OE) congener and closed-ecosystem (CE) congener] and study genus [*Parinari* (Chrysobalanaceae)*, Ozoroa* (Anacardiaceae), *Syzygium* (Myrtaceae) and *Lannea* (Anacardiaceae)] per environmental variable of mean annual temperature (MAT), temperature seasonality (TS), annual precipitation (AP), precipitation seasonality (PS), dry season precipitation (DS), frost, fire, herbivory (Hrbv) and the topographic wetness index (TWI).

mcad124_suppl_Supplementary_Material
