# Supplementary material for: Underground trees inhabit varied environmental extremes across the Afrotropics
Source: Ann Bot. 2023 Aug 29;133(5-6):757–72. doi: 10.1093/aob/mcad124 (PMC11082510; doi:10.1093/aob/mcad124)
Supplement: mcad124_suppl_Supplementary_Material [file mcad124_suppl_supplementary_material.docx]

Supplementary Information for

**Underground trees inhabit varied environmental extremes across the Afrotropics**

Anya P. Courtenay,* Peter W. Moonlight, R. Toby Pennington, Caroline E.R. Lehmann

*Corresponding author email: [a.courtenay@ed.ac.uk](mailto:a.courtenay@ed.ac.uk)

Additional content includes maps of occurrence records (Figures S1-S4) and sampling density (Figure S5) as well as the Topographic Wetness Index dataset (Figure S6) alongside a methodology for how it was derived (Method S1). Also presented are a correlation matrix of environmental variables (Table S1), mean environmental values (Table S2) and box plots of study taxa range sizes (Figure S7).


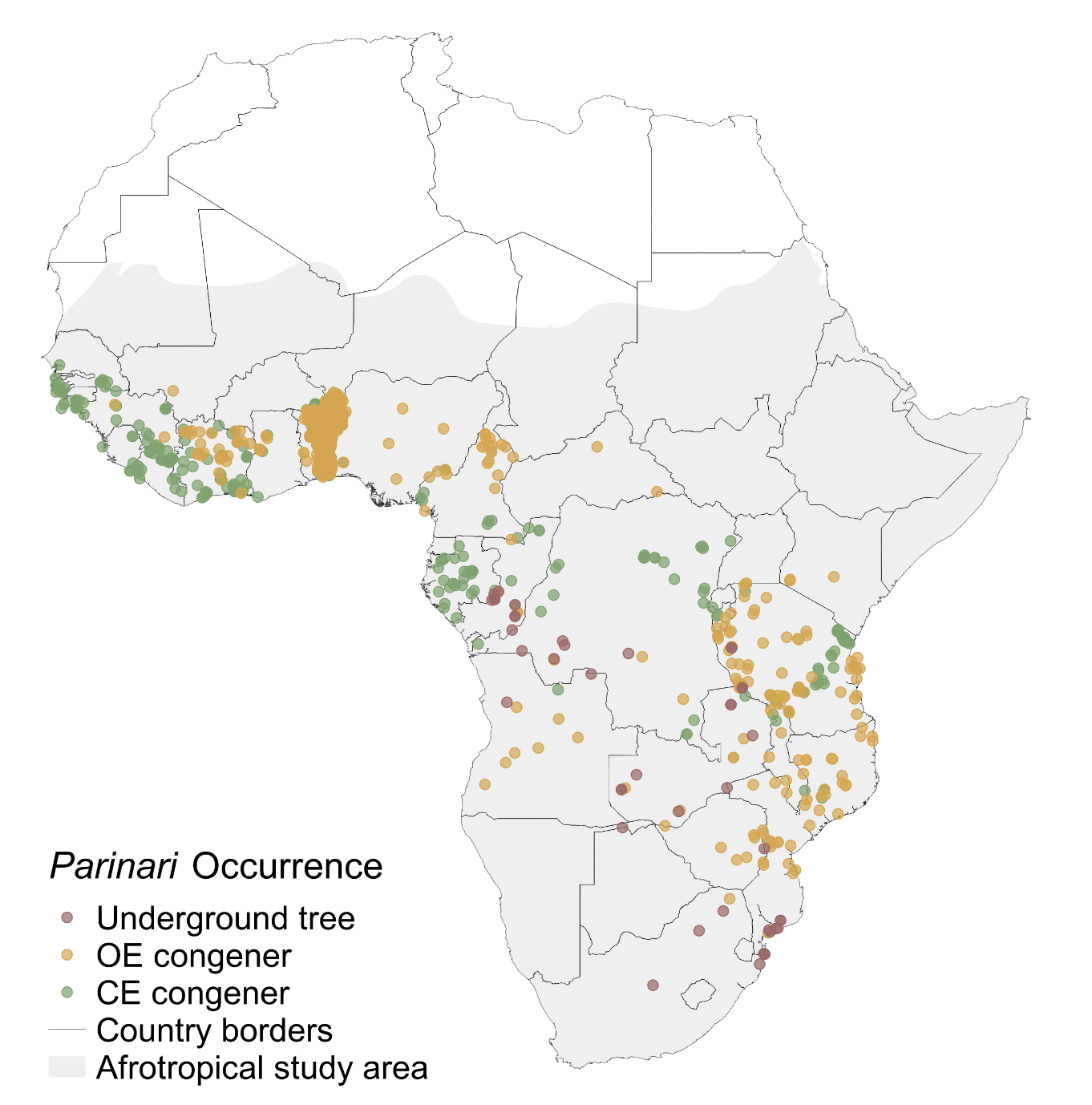


**Figure S1.** Map of cleaned GBIF occurrence records in the Afrotropical study region for *Parinari* (Chrysobalanaceae) underground trees, open ecosystem (OE) congeners and closed ecosystem (CE) congeners.


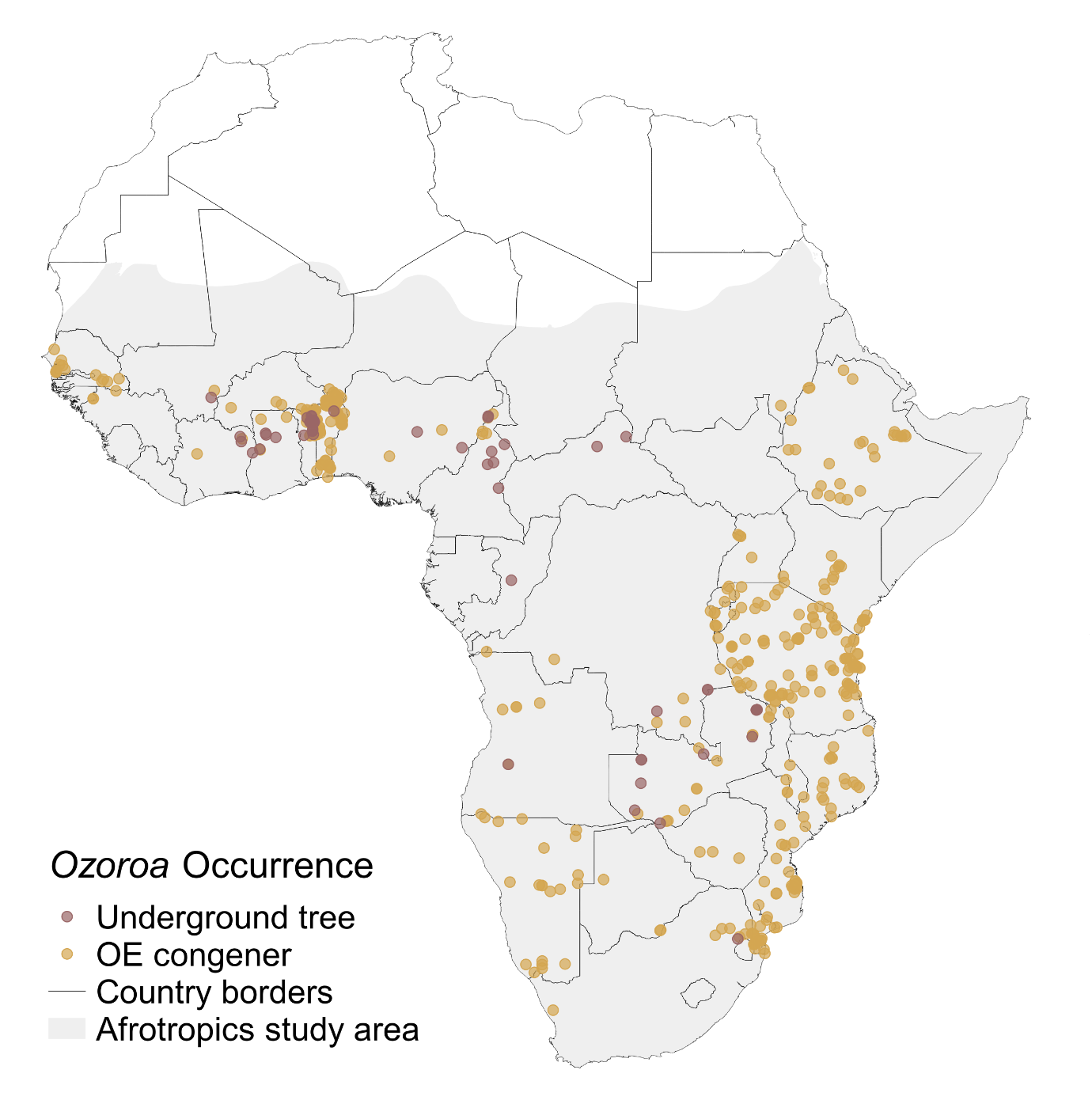


**Figure S2.** Map of cleaned GBIF occurrence records in the Afrotropical study region for *Ozoroa* (Anacardiaceae) underground trees, open ecosystem (OE) congeners and closed ecosystem (CE) congeners.


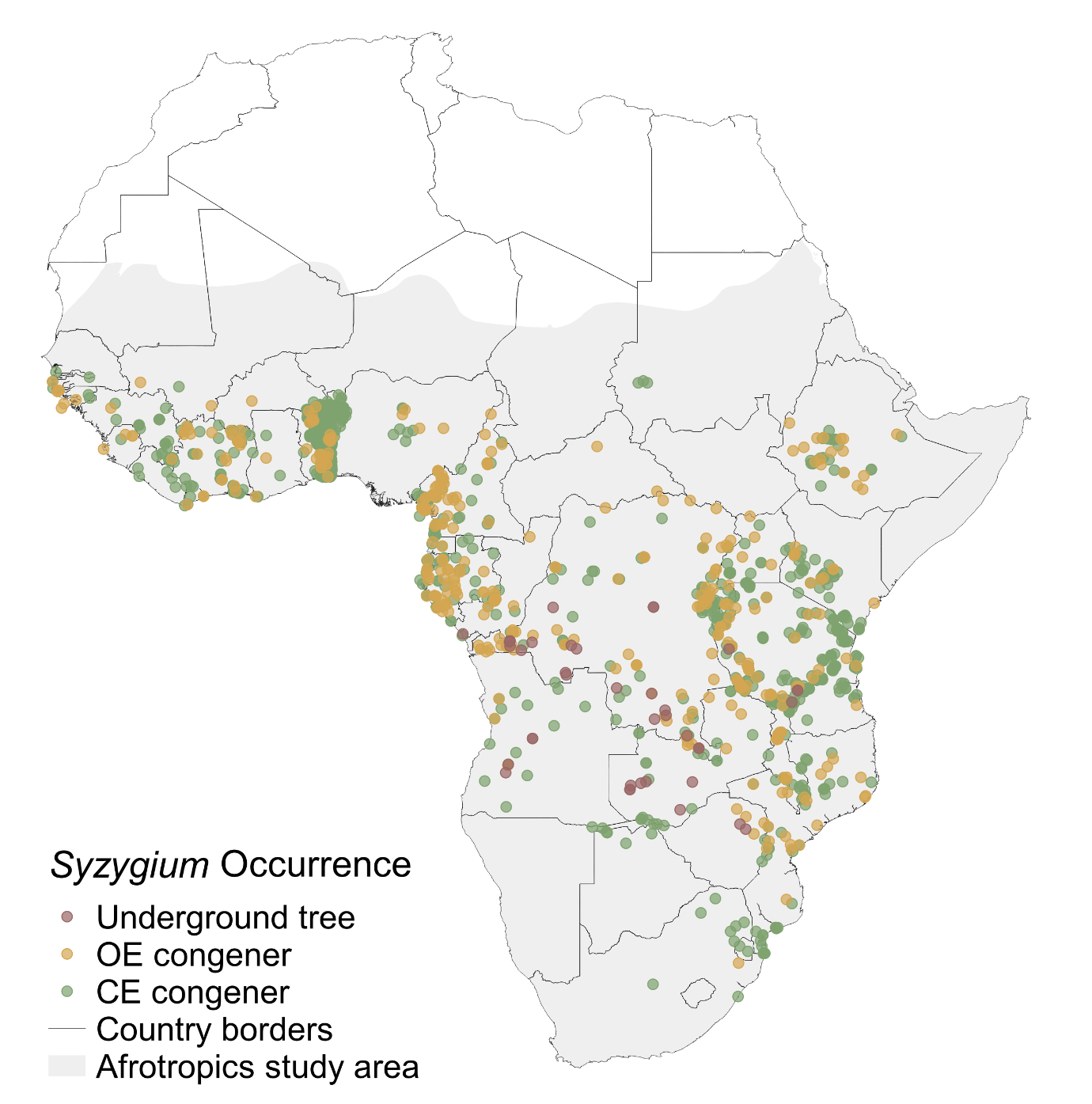


**Figure S3.** Map of cleaned GBIF occurrence records in the Afrotropical study region for *Syzygium* (Myrtaceae) underground trees, open ecosystem (OE) congeners and closed ecosystem (CE) congeners.


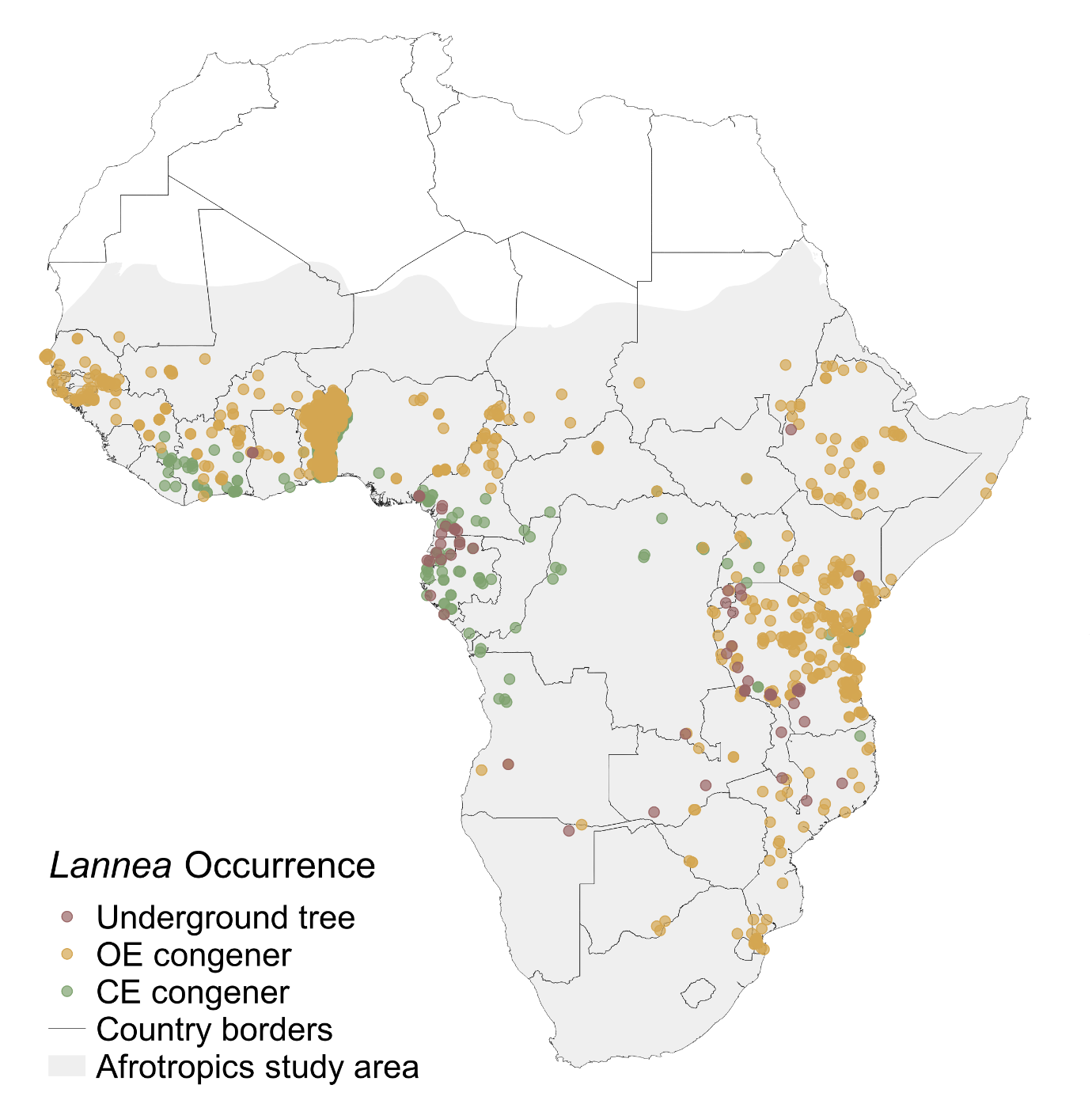


**Figure S4.** Map of cleaned GBIF occurrence records in the Afrotropical study region for *Lannea* (Anacardiaceae) underground trees, open ecosystem (OE) congeners and closed ecosystem (CE) congeners.


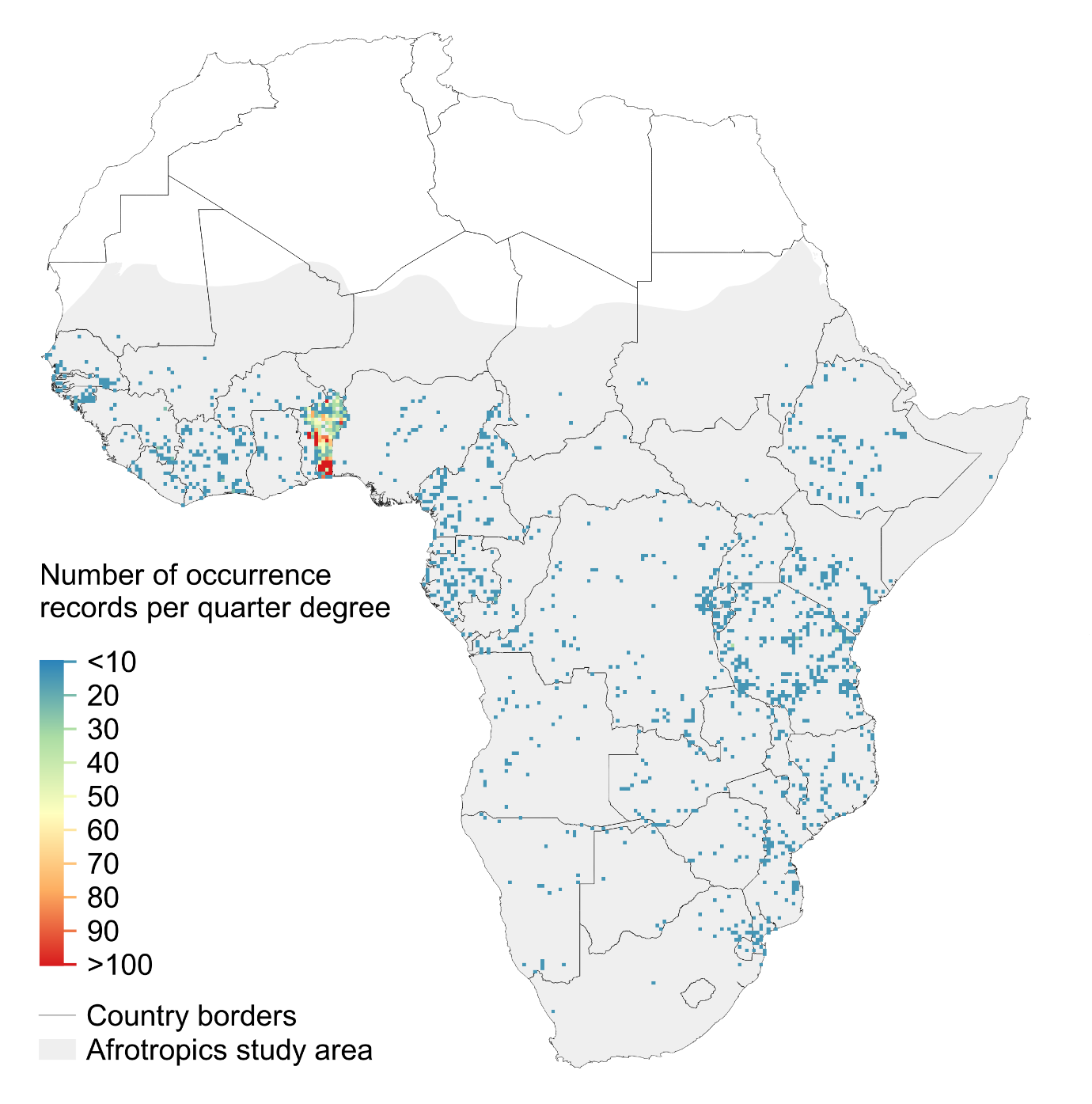


**Figure S5**. Map showing sampling density as the number of cleaned GBIF occurrence records for all georeferenced taxa in the genera *Parinari* (Chrysobalanaceae)*,* *Ozoroa* (Anacardiaceae), *Syzygium* (Myrtaceae) and *Lannea* (Anacardiaceae) per quarter degree grid square across the Afrotropics.

**Method S1.** Process used to derive a Topographic Wetness Index for the African continent

Reliable soil data at a continental scale are difficult to link to underground tree occurrence data (Meller *et al.*, 2022). However, soil characteristics are also known to be important to the limits of underground trees through a complex of drainage, grain size and soil depth (White, 1976). The catena concept (Brunner *et al.*, 2004) demonstrates topographic variation in soil properties from more earthy and sandy at higher elevations, down to greater clay contents in depressions where water accumulates (Borden *et al*., 2020). The way in which soil moisture is facilitated spatiotemporally along topographic gradients positions plants within a landscape. Further, soil moisture mediates climate patterns and the availability of water to plants (Breshears *et al*., 2009); making it one of the most important determinants of vegetation composition (Kopechký *et al*., 2021). Despite this importance, soil moisture is difficult to measure and interpolate across large spatial and temporal scales (Kopechký *et al*., 2021). Hence, particular consideration in these analyses was given to the processing and inclusion of hillslope hydrology factors that can account for important variation in soil properties.

The Topographic Wetness Index (TWI) is a proxy for soil moisture that can be derived from Digital Elevation Models (DEMs). Previously, TWI has been linked to patterns of plant species richness (Sørensen *et al*., 2006) and biomass (Xu *et al.*, 2015). TWI also represents the influence of topography, with catenary variation (Sørensen *et al*., 2006) and other soil properties significant to plants such as the distribution of organic matter (Pei *et al*., 2010) and soil carbon (Sumfleth and Duttmann, 2008). TWI is a physically-based model of catchment parameters derived from slope that calculates water supply from the contributing hillslope by routing it as drainage down the channel network through all cells in a DEM (Beven and Kirkby, 1979). The TWI quantifies the balance between water accumulation and drainage at a local catchment scale, using the following equation: *TWI = ln(upslope area / tan(slope))*.

As a comprehensive metric of soil moisture content and saturated areas, TWI was used as an indicator of the waterlogging with which some underground trees have been associated (White, 1976; Maurin *et al.*, 2014; Zigelski *et al*., 2018). To create a raster layer of TWI for the African continent, a four-step hydro-analysis process was implemented, outlined by Mattivi *et al.* (2019), using the R package ‘whitebox tools’ (Lindsay, 2016). Starting with a DEM sourced from the SRTM (2013) and maintaining a 30 m resolution, the GIS layer was hydrologically corrected by filling sinks/depressions. Secondly, flow accumulation was delineated from the contributing area using the D8 flow routing algorithm, which determines the single direction of flow to the steepest downslope neighbouring cell. Next, slope was derived in degrees from the DEM. Based on the TWI equation and using the *Wetness Index* function, slope was combined with flow accumulation to calculate TWI. The final product, a raster layer with a TWI value per 30 m pixel across Africa (<https://doi.org/10.5281/zenodo.7875980>) was included as a parameter for waterlogging in analyses.


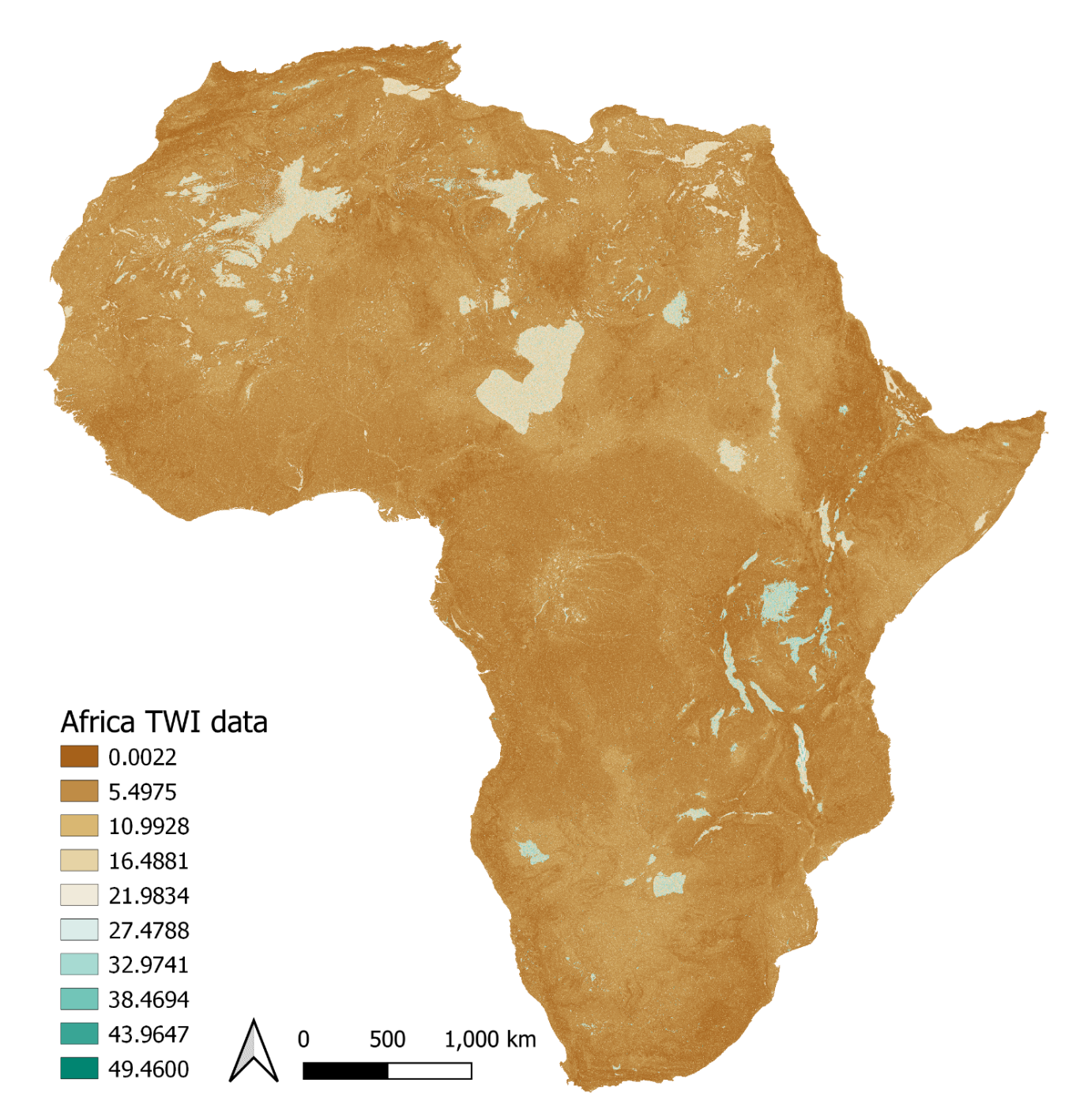


**Figure S6.** A map of the Topographic Wetness Index (TWI) spatial database created for Africa in this study.

**Table S1.** A correlation matrix confirming no autocorrelation (all values < 0.7) among standardised bioclimatic and environmental stress variables used in this study: mean annual temperature (MAT), temperature seasonality (TS), annual precipitation (AP), precipitation seasonality (PS), dry season precipitation (DP), frost, fire, herbivory (Hrbv) and the Topographic Wetness Index (TWI).

|  | MAT | TS | AP | PS | DP | Fire | Frost | TWI | Hrbv |
| --- | --- | --- | --- | --- | --- | --- | --- | --- | --- |
| MAT |  | -0.2001 | -0.0833 | 0.3219 | -0.1320 | 0.0688 | -0.2201 | 0.1988 | -0.0757 |
| TS | -0.2001 |  | -0.6390 | 0.4076 | -0.4248 | -0.1136 | -0.4315 | 0.1321 | 0.2625 |
| AP | -0.0833 | -0.6390 |  | -0.5285 | 0.5751 | 0.1143 | 0.4505 | -0.2170 | -0.2718 |
| PS | 0.3219 | 0.4076 | -0.5285 |  | -0.6828 | 0.0647 | -0.3430 | 0.1729 | 0.1792 |
| DP | -0.1320 | -0.4248 | 0.5751 | -0.6828 |  | -0.2059 | 0.2270 | -0.0923 | -0.1996 |
| Fire | 0.0688 | -0.1136 | 0.1143 | 0.0647 | -0.2059 |  | 0.1862 | -0.0199 | 0.0483 |
| Frost | -0.2201 | -0.4315 | 0.4505 | -0.3430 | 0.2270 | 0.1862 |  | -0.1139 | -0.1120 |
| TWI | 0.1988 | 0.1321 | -0.2170 | 0.1729 | -0.0923 | -0.0199 | -0.1139 |  | 0.0788 |
| Hrbv | -0.0757 | 0.2625 | -0.2718 | 0.1792 | -0.1996 | 0.0483 | -0.1120 | 0.0788 |  |

**Table S2.** Mean values and standard deviation for occurrence data of each habit/habitat category (underground tree, open ecosystem (OE) congener and closed ecosystem (CE) congener) and study genus (*Parinari* (Chrysobalanaceae)*,* *Ozoroa* (Anacardiaceae), *Syzygium* (Myrtaceae) and *Lannea* (Anacardiaceae) per environmental variable of mean annual temperature (MAT), temperature seasonality (TS), annual precipitation (AP), precipitation seasonality (PS), dry season precipitation (DS), frost, fire, herbivory (Hrbv) and the Topographic Wetness Index (TWI).

|  | | **MAT**  **(°C)** | **TS**  **(°C)** | **AP**  **(mm)** | **PS**  **(coefficient)** | **DP**  **(mm)** | **Frost**  **(mean annual days)** | **Fire**  **(km^2^)** | **Hrbv**  **(kg/km^2^)** | **TWI** |
| --- | --- | --- | --- | --- | --- | --- | --- | --- | --- | --- |
| ***Parinari*** | Underground tree | 27.57  ± 2.74 | 230.18  ± 49.36 | 952.75  ± 222.00 | 115.29  ± 9.08 | 43.11  ± 40.40 | 48.57  ± 13.63 | 0.33  ± 0.31 | 1053.80  ± 1248.38 | 5.31  ± 2.98 |
|  | OE congener | 26.26  ± 1.81 | 147.92  ± 28.60 | 1192.06  ± 205.78 | 77.17  ± 14.94 | 27.01  ± 19.14 | 72.88  ± 9.28 | 0.22  ± 0.24 | 593.95  ± 433.33 | 5.29  ± 2.36 |
|  | CE congener | 25.32  ± 3.41 | 152.47  ± 76.30 | 1546.97  ± 669.52 | 81.88  ± 29.50 | 64.31  ± 90.50 | 62.59  ± 14.82 | 0.24  ± 0.40 | 805.94  ± 629.91 | 6.26  ± 4.60 |
| ***Ozoroa*** | Underground tree | 24.50  ± 2.65 | 177.65  ± 46.40 | 1257.10  ± 360.85 | 93.37  ± 14.75 | 10.446  ± 23.68 | 64.31  ± 13.49 | 0.43  ± 0.39 | 945.77  ± 628.74 | 4.66  ± 2.12 |
|  | OE congener | 24.57  ± 3.73 | 183.12  ± 72.41 | 1022.67  ± 295.65 | 93.68  ± 20.06 | 21.40  ± 31.34 | 60.35  ± 16.50 | 0.29  ± 0.38 | 1431.80  ± 1163.47 | 5.30  ± 3.21 |
| ***Syzygium*** | Underground tree | 21.41  ± 2.58 | 135.26  ± 79.66 | 1339.24  ± 328.34 | 86.25  ± 24.68 | 28.07  ± 53.32 | 83.19  ± 4.02 | 0.26  ± 0.36 | 644.37  ± 675.35 | 6.43  ± 5.83 |
|  | OE congener | 22.42  ± 3.50 | 124.39  ± 60.13 | 1713.70  ± 906.77 | 74.58  ± 21.18 | 64.58  ± 78.12 | 71.69  ± 16.34 | 0.18  ± 0.32 | 688.95  ± 567.88 | 4.94  ± 3.74 |
|  | CE congener | 23.41  ± 4.38 | 152.82  ± 63.00 | 1396.53  ± 614.86 | 82.70  ± 21.59 | 49.44  ± 77.26 | 68.20  ± 13.03 | 0.24  ± 0.35 | 977.60  ± 736.62 | 5.46  ± 4.12 |
| ***Lannea*** | Underground tree | 20.77  ± 3.31 | 126.07  ± 61.70 | 1591.19  ± 838.83 | 81.98  ± 22.56 | 70.27  ± 94.45 | 80.04  ± 8.61 | 0.11  ± 0.25 | 746.55  ± 483.95 | 4.29  ± 4.02 |
|  | OE congener | 26.61  ± 2.16 | 175.93  ± 49.09 | 1109.94  ± 279.06 | 91.20  ± 18.30 | 16.851  ± 20.95 | 63.01  ± 14.49 | 0.31  ± 0.33 | 958.37  ± 632.04 | 5.37  ± 2.82 |
|  | CE congener | 26.84  ± 0.82 | 164.85  ± 28.42 | 1153.79  ± 242.25 | 88.07  ± 14.78 | 17.06  ± 27.67 | 70.81  ± 7.10 | 0.23  ± 0.24 | 718.15  ± 297.00 | 5.21  ± 2.12 |


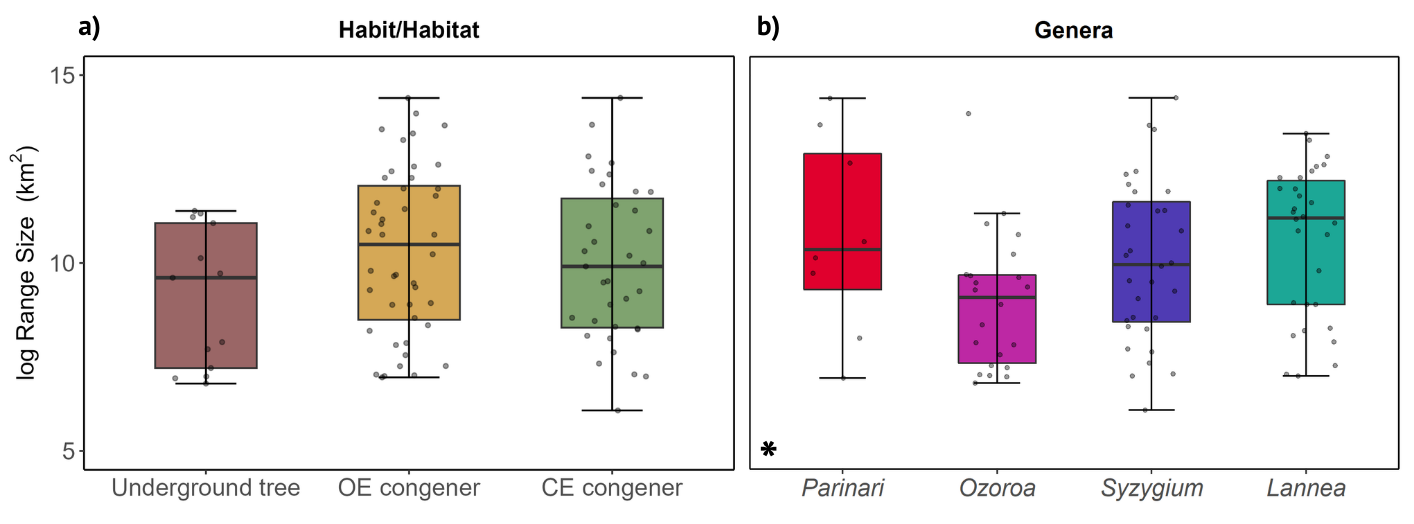


**Figure S7****.** Logged extent of occurrence (EOO) range size for a) taxa of all genera as underground trees, open ecosystem congeners (OE congener) and closed ecosystem congeners (CE congener); and b) all taxa by genus with significant results (* *P* < 0.05) from a one-way ANOVA.

**Literature Cited in Supplementary Information**

Beven, K.J. and Kirkby, M.J. (1979) ‘A physically based, variable contributing area model of basin hydrology / Un modèle à base physique de zone d’appel variable de l’hydrologie du bassin versant’, *Hydrological Sciences Bulletin*, 24(1), pp. 43–69. Available at: https://doi.org/10.1080/02626667909491834.

Borden, R.W., Baillie, I.C. and Hallett, S.H. (2020) ‘The East African contribution to the formalisation of the soil catena concept’, *CATENA*, 185, p. 104291. Available at: https://doi.org/10.1016/j.catena.2019.104291.

Breshears, D.D., Myers, O.B. and Barnes, F.J. (2009) ‘Horizontal heterogeneity in the frequency of plant-available water with woodland intercanopy–canopy vegetation patch type rivals that occuring vertically by soil depth’, *Ecohydrology*, 2(4), pp. 503–519. Available at: https://doi.org/10.1002/eco.75.

Brunner, A.C., Park, S.J., Ruecker, G.R., Dikau, R. and Vlek, P.L.G. (2004) ‘Catenary soil development influencing erosion susceptibility along a hillslope in Uganda’, *CATENA*, 58(1), pp. 1–22. Available at: https://doi.org/10.1016/j.catena.2004.02.001.

Kopechký, M., Macek, M. and Wild, J. (2021) ‘Topographic Wetness Index calculation guidelines based on measured soil moisture and plant species composition’, *Science of The Total Environment*, 757, p. 143785. Available at: https://doi.org/10.1016/j.scitotenv.2020.143785.

Lindsay, J.B. (2016) ‘Whitebox GAT: A case study in geomorphometric analysis’, *Computers & Geosciences*, 95, pp. 75–84. Available at: https://doi.org/10.1016/j.cageo.2016.07.003.

Mattivi, P., Franci, F., Lambertini, A. and Bitelli, G. (2019) ‘TWI computation: a comparison of different open source GISs’, *Open Geospatial Data*, Software and Standards, 4(1), p. 6. Available at: https://doi.org/10.1186/s40965-019-0066-y.

Maurin, O., Davies, T.J., Burrows *et al*. (2014) ‘Savanna fire and the origins of the “underground forests” of Africa’, *New Phytologist*, 204(1), pp. 201–214. Available at: https://doi.org/10.1111/nph.12936.

Meller, P., Stellmes, M., Fidelis, A. and Finckh, M. (2022) ‘Correlates of geoxyle diversity in Afrotropical grasslands’, *Journal of Biogeography*, 49(2), pp. 339–352. Available at: https://doi.org/10.1111/jbi.14305.

Sørensen, R., Zinko, U. and Seibert, J. (2006) ‘On the calculation of the topographic wetness index: evaluation of different methods based on ﬁeld observations’, *Hydrology and Earth System Sciences*, 10(1), p. 12.

SRTM (2013) *Shuttle Radar Topography Mission (SRTM) Global. Distributed by OpenTopography* [Preprint]. Available at: https://doi.org/10.5069/G9445JDF.

Sumfleth, K. and Duttmann, R. (2008) ‘Prediction of soil property distribution in paddy soil landscapes using terrain data and satellite information as indicators’, *Ecological Indicators*, 8(5), pp. 485–501. Available at: https://doi.org/10.1016/j.ecolind.2007.05.005.

White, F. (1976) ‘The underground forests of Africa: a preliminary review’, *Garden’s Bulletin*.

Xu, Y., Franklin S.B, Qinggang, W. *et al.* (2015) ‘Topographic and biotic factors determine forest biomass spatial distribution in a subtropical mountain moist forest’, *Forest Ecology and Management*, 357, pp. 95–103. Available at: https://doi.org/10.1016/j.foreco.2015.08.010.

Zigelski, P., Lages, F. and Finckh, M. (2018) ‘Seasonal changes of biodiversity patterns and habitat conditions in a flooded savanna - the Cameia National Park Biodiversity Observatory in the Upper Zambezi catchment, Angola.’, *Biodiversity and Ecology*, 6, pp. 438–447.
